# m^1^A in CAG repeat RNA binds to TDP-43 and induces neurodegeneration

**DOI:** 10.1038/s41586-023-06701-5

**Published:** 2023-11-08

**Authors:** Yuxiang Sun, Hui Dai, Xiaoxia Dai, Jiekai Yin, Yuxiang Cui, Xiaochuan Liu, Gwendolyn Gonzalez, Jun Yuan, Feng Tang, Nan Wang, Alexandra E. Perlegos, Nancy M. Bonini, X. William Yang, Weifeng Gu, Yinsheng Wang

**Affiliations:** 1 Department of Chemistry, University of California Riverside, Riverside, CA USA; 2 Department of Molecular, Cell and Systems Biology, University of California Riverside, Riverside, CA USA; 3 Environmental Toxicology Graduate Program, University of California Riverside, Riverside, CA USA; 4 Center for Neurobehavioral Genetics, The Jane and Terry Semel Institute for Neuroscience and Human Behavior, University of California Los Angeles, Los Angeles, CA USA; 5 Neurosciences Graduate Group, University of Pennsylvania, Philadelphia, PA USA; 6 Department of Biology, University of Pennsylvania, Philadelphia, PA USA; 7 Department of Psychiatry and Biobehavioral Sciences, David Geffen School of Medicine, University of California Los Angeles, Los Angeles, CA USA

**Keywords:** RNA, Molecular neuroscience, Huntington's disease, Neurodegeneration, Biophysical chemistry

## Abstract

Microsatellite repeat expansions within genes contribute to a number of neurological diseases^1,2^. The accumulation of toxic proteins and RNA molecules with repetitive sequences, and/or sequestration of RNA-binding proteins by RNA molecules containing expanded repeats are thought to be important contributors to disease aetiology^3–9^. Here we reveal that the adenosine in CAG repeat RNA can be methylated to *N*^1^-methyladenosine (m^1^A) by TRMT61A, and that m^1^A can be demethylated by ALKBH3. We also observed that the m^1^A/adenosine ratio in CAG repeat RNA increases with repeat length, which is attributed to diminished expression of ALKBH3 elicited by the repeat RNA. Additionally, TDP-43 binds directly and strongly with m^1^A in RNA, which stimulates the cytoplasmic mis-localization and formation of gel-like aggregates of TDP-43, resembling the observations made for the protein in neurological diseases. Moreover, m^1^A in CAG repeat RNA contributes to CAG repeat expansion-induced neurodegeneration in *Caenorhabditis elegans* and *Drosophila*. In sum, our study offers a new paradigm of the mechanism through which nucleotide repeat expansion contributes to neurological diseases and reveals a novel pathological function of m^1^A in RNA. These findings may provide an important mechanistic basis for therapeutic intervention in neurodegenerative diseases emanating from CAG repeat expansion.

## Abstract (web summary)

TDP-43 binds to *N*^1^-methyladenosine on CAG repeat RNA, resulting in the formation of gel-like TDP-43 aggregates in the cytoplasm that resemble those observed in neurological disease pathology.

## Main

Nucleotide repeat expansions contribute to a number of neurological diseases^1,2^. For instance, expansion of a GGGGCC hexanucleotide repeat in the *C9ORF72* gene is the major genetic cause of amyotrophic lateral sclerosis^10^ (ALS), and CAG repeat expansions contribute to the development of multiple neurodegenerative disorders including Huntington’s disease and various forms of spinocerebellar ataxia^2^ (SCA). Several mechanisms have been proposed for neurological diseases elicited by repeat expansions; these include neurotoxicity arising from formation of neuronal intranuclear inclusions and amyloid-like aggregates of proteins translated from repeat RNAs^3,4^, and the ensuing impairment of the ubiquitin–proteasome system^11^. Additionally, accumulation and aberrant phase separation of toxic RNAs, and their sequestration of RNA-binding proteins are also important contributors to disease aetiology^5–9^.

Cytoplasmic mis-localization and aggregation of TDP-43 in the degenerated regions of the brain are clinical hallmarks of many neurological diseases, including ALS, frontotemporal lobar degeneration^12,13^ (FTLD), and CAG repeat expansion disorders, such as Huntington’s disease^14^ and SCA^15^. Additionally, intermediate-length CAG repeat expansions in the *ATXN2* gene are significantly associated with ALS^16^, in which genetic depletion of *ATXN2* extends lifespan and mitigates pathological aggregates in TDP-43 transgenic mice^15^. TDP-43 contains two RNA-recognition motifs (RRMs) and a C-terminal low-complexity domain^13^ (LCD), through which the protein can undergo intermolecular interactions and assemble into phase-separated liquid condensates^17^. Furthermore, phase-separated droplets of RNA–protein complexes can become less dynamic and aggregate over time, suggesting that aberrant phase transition contributes to the aggregation of RNA-binding proteins^18^.

Dynamic post-transcriptional modifications in RNA, especially methylation at the *N*^6^ position of adenosine, constitute an important mechanism regulating the stability and translation efficiency of mRNAs^19,20^, and dysregulation of this methylation in mRNAs are linked with human diseases^21^. Here we show that CAG repeat expansions lead to increased levels of m^1^A, which binds to TDP-43, alters its subcellular distribution and phase separation behaviour, and contributes to neurodegeneration.

## m^1^A increases with CAG repeat length

Previous studies documented that the age of onset of neurological disorders arising from CAG repeat expansion is negatively correlated with repeat length, whereas disease severity is positively associated with repeat length^2^. We set out to test whether methylated adenosines—especially *N*^6^-methyladenosine (m^6^A) and m^1^A—are present in CAG repeat RNA and if so, how their modification frequencies may be influenced by repeat length and how they modulate RNA–protein interactions.

**Fig. 1:**
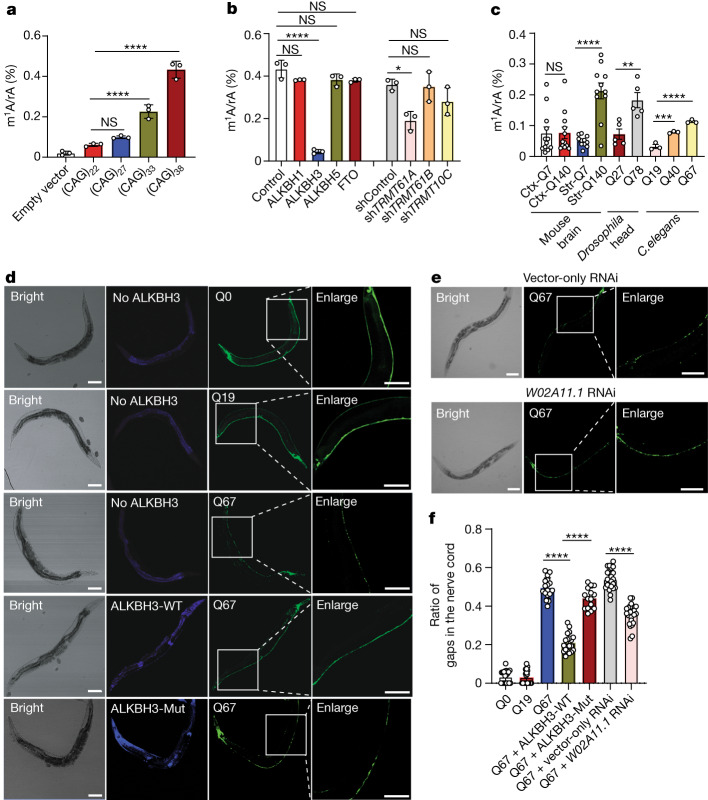
m^1^A in CAG repeat RNA increases with repeat length and contributes to neurodegeneration in *C. elegans*. **a**–**c**, The m^1^A/rA ratios in ectopically expressed CAG repeat RNAs isolated from HEK293T cells (**a**; *n* = 3 biologically independent experiments), ectopically expressed (CAG)_38_ RNA isolated from HEK293T cells with ectopic co-expression of the indicated RNA demethylases or stable knockdown of m^1^A methyltransferases (**b**; *n* = 3 biologically independent experiments), and CAG repeat RNA from mouse brain (*n* = 11 and 12 biologically independent samples for striatum (str) and cortex (ctx) tissues, respectively), *Drosophila* head (*n* = 5 biologically independent samples, 10-day old males, 30 heads per sample) and *C. elegans* (*n* = 3 biologically independent experiments) (**c**). **d**,**e**, Fluorescence images of representative Q0, Q19 and Q67 nematodes, in Q67 nematodes with ectopic expression of wild-type human ALKBH3 (ALKBH3-WT) or its catalytically inactive mutant (ALKBH3-Mut) (**d**), and in Q67 nematodes with or without knockdown of *W02A11.1* gene (**e**). **f**, Ratios of gaps in the nerve cord in Q67 worms with or without expression of human ALKBH3-WT and ALKBH3-Mut (Q0: *n* = 20 worms; Q19: *n* = 20 worms; Q67: *n* = 17 worms; Q67 + ALKBH3-WT: *n* = 20 worms; Q67 + ALKBH3-Mut: *n* = 15 worms), and in Q67 nematodes with or without knockdown of *W02A11.1* gene (Q67 + vector RNAi: *n* = 22 worms; Q67 + *W02A11.1* RNAi: *n* = 23 worms). The ratios of gaps were calculated by dividing the total length of gaps over the entire length of the nerve cord. Data are mean ± s.d. *P* values for the data of mouse brain and *Drosophila* head samples in **c**, and between Q67 + vector RNAi and Q67 + *W02A11.1* RNAi in **f** were determined using two-tailed Student’s *t*-test; all other *P* values were determined using one-way ANOVA with Tukey’s multiple comparisons test. NS, not significant (*P* > 0.05). **b**, *P = 0.021. **c**, **P = 0.0058 and ***P = 0.0007. ****P < 0.0001. Scale bars, 100 μm. Source Data

To address these questions, we ectopically expressed mRNAs with different CAG repeat lengths in HEK293T cells, isolated the CAG repeat RNA and quantified the levels of m^1^A and m^6^A in the resulting RNA samples using liquid chromatography–tandem mass spectrometry^22^ (LC–MS/MS). The results revealed a progressive increase in the frequency of m^1^A (relative to adenosine (rA)) with repeat length, which is accompanied with a gradual diminution in m^6^A level (Fig. 1a and Supplementary Figs. 2, 3 and 4a). Of note, the level of m^1^A in (CAG)_38_ RNA was even higher than that of m^6^A (Fig. 1a and Supplementary Fig. 4a). We also annealed the purified CAG repeat RNA with two oligodeoxynucleotides whose sequences are complementary to the 5′ and 3′ flanking sequences of the (CAG)_38_ RNA, and removed the RNA in the ensuing RNA/DNA hybrid with RNase H. The results showed that such removal led to an increased level of m^1^A, but not m^6^A, underscoring a higher frequency of m^1^A in (CAG)_38_ repeat than in flanking sequences (Supplementary Fig. 5a,b). Notably, (CAG)_22_ and (CAG)_38_ RNAs were expressed at similar levels (Supplementary Fig. 5c).

m^1^A can be demethylated by ALKBH1 and ALKBH3^23,24^, and m^6^A can be demethylated by FTO and ALKBH5^25,26^. We tested whether these demethylases could also act on m^6^A and m^1^A in CAG repeat RNAs. We observed a marked attenuation (by approximately tenfold) in the levels of m^1^A in (CAG)_38_ after ectopic expression of ALKBH3, but not ALKBH1, FTO or ALKBH5 (Fig. 1b). In addition, we found that m^6^A in (CAG)_38_ could be demethylated by FTO (Supplementary Fig. 4b), but not by ALKBH1 or ALKBH5; ectopic expression of ALKBH3 also led to a diminished level of m^6^A in (CAG)_38_ RNA (Supplementary Fig. 4b).

Several m^1^A methyltransferases have been identified, including TRMT6–TRMT61A^27^, TRMT61B^28^ and TRMT10C^29^. Although TRMT61B and TRMT10C are involved in m^1^A formation in mitochondrial tRNAs^28,29^, the TRMT6–TRMT61A heterodimer can install m^1^A in both tRNAs and mRNAs^27,30^. TRMT61A is the catalytic subunit of the methyltransferase complex and it is highly conserved from invertebrates to humans^27^. We therefore used short hairpin RNA (shRNA) to knock down TRMT61A expression in human cells (Supplementary Fig. 6a,d) and measured the levels of m^1^A in (CAG)_38_ RNA. TRMT61A depletion led to a significant decrease in m^1^A level in (CAG)_38_ RNA, whereas we observed no change in m^6^A level (Fig. 1b and Supplementary Figs. 4b and 6e,f). We also observed that recombinant TRMT6–TRMT61A proteins could catalyse the formation of m^1^A in (CAG)_16_ RNA in vitro, whereas the heat-inactivated enzyme did not (Supplementary Fig. 7). Knockdown of TRMT61B or TRMT10C in HEK293T cells, however, did not alter the level of m^1^A in (CAG)_38_ RNA (Fig. 1b).

We next examined whether the frequency of m^1^A also increases with CAG repeat length in vivo. We used affinity purification to isolate CAG repeat RNA from the striatum and cortex tissues of wild-type mice that carry 7 CAG repeats (Q7) or transgenic mice expressing one wild-type endogenous *Htt* allele and a second *Htt* allele with knock-in of human mutant *HTT* (*mHTT*) exon 1, which contains 140 CAG repeats^31^ (Q140). LC–MS/MS analysis showed that the m^1^A level was significantly higher in Q140 mRNA than in Q7 mRNA isolated from striatal tissues (Fig. 1c), but there was no significant difference in m^6^A level in Q140 and Q7 mRNA (Supplementary Fig. 4c). In addition, no apparent difference was detected for the level of m^6^A or m^1^A in the cortical tissues of mice expressing the two different lengths of CAG repeat. These observations parallel the previous finding that repeat length-dependent transcriptional signatures are more prominent in the striatum than in the cortex tissues of these mice^31^. Similarly, m^1^A, but not m^6^A, was present at a higher level in CAG repeat mRNA isolated from *Drosophila* heads with neuronal-specific expression of an *SCA3* transgene with 78 repeats of CAG (Q78), than those with a short Q27 CAG repeat^6^ (Fig. 1c and Supplementary Fig. 4c). We also detected markedly increased levels of m^1^A in CAG repeat RNA isolated from *C. elegans* with pan-neuronal expression of (CAG)_40_ and (CAG)_67_ mRNA (Q40 and Q67) than in worms expressing (CAG)_19_ mRNA^32^ (Q19), although no difference was detected in the levels of m^6^A in these mRNA samples (Fig. 1c and Supplementary Fig. 4c).

We next examined the role of TRMT61A in modulating m^1^A level in CAG repeat RNA in *C. elegans*. We performed RNA-mediated interference (RNAi) targeting the *W02A11.1* gene, which is the nematode orthologue of the human *TRMT61A* gene. Knockdown of *W02A11.1* led to a significantly diminished level of m^1^A in CAG repeat RNA extracted from the nematodes, whereas there was no significant difference in m^6^A level (Supplementary Fig. 8a,b). Together, these results indicate that the levels of m^1^A in CAG repeat RNA increase with repeat length, with the m^1^A in CAG repeat RNA being installed and removed by TRMT61A and ALKBH3, respectively.

## Diminished ALKBH3 leads to increased m^1^A

**Extended Data Fig. 1:**
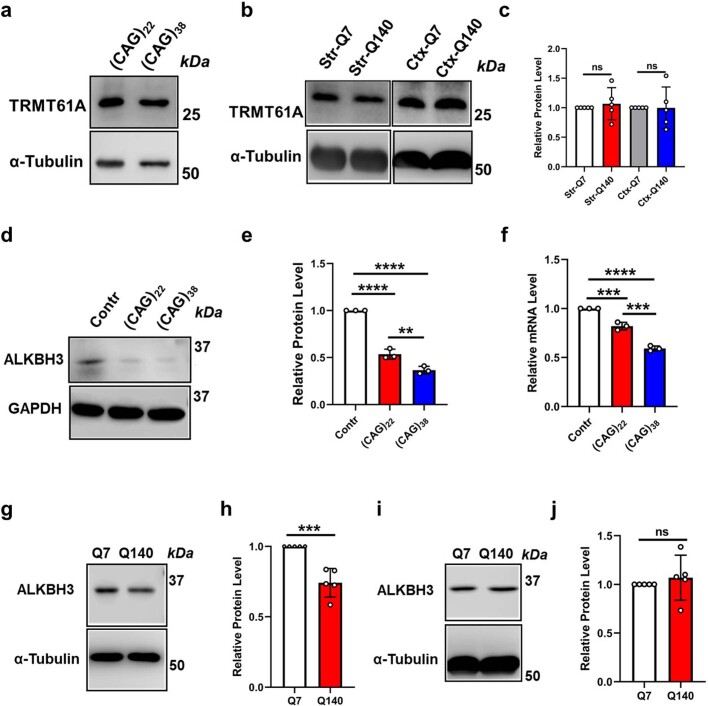
Expression of CAG repeat RNA suppresses the expression of ALKBH3, but not TRMT61A in cells and striatum tissues of mice. **a**-**c**, Western blot images and quantification results showing the relative expression levels of TRMT61A in HEK293T cells with ectopic expression of (CAG)_22_ and (CAG)_38_ RNA (**a**) and in striatum (Str) and cortex (Ctx) tissues of Q7 and Q140 mice (**b**, **c**). n = 5 biologically independent samples. Data are mean ± s.d. *P* values were determined using Two-tailed Student’s *t*-test. **d**-**e**, Western blot images and quantification results showing the relative expression levels of ALKBH3 in HEK293T cells with ectopic expression of (CAG)_22_ and (CAG)_38_ RNA. **f**, RT-qPCR results showing the relative expression levels of ALKBH3 mRNA in HEK293T cells with ectopic expression of (CAG)_22_ and (CAG)_38_ RNA, or with empty plasmid control (Contr). Data in **e**-**f** are mean ± s.d., and represent three biologically independent experiments. *P* values were determined using one-way ANOVA with Tukey’s multiple comparisons test. ns, *P* > 0.05; **P = 0.0041 in **e**; ***P = 0.0005 between Contr and (CAG)_22_, and ***P = 0.0001 between (CAG)_22_ and (CAG)_38_ in **f**; ****P < 0.0001. **g**-**j**, Western blot images and quantification results showing the relative expression levels of ALKBH3 protein in striatum (**g**-**h**) and cortex (**i**-**j**) tissues of Q7 and Q140 mice (n = 5 biologically independent samples of striatum or cortex). The loading control was detected in a separate gel in parallel for **d** and **g**. Data are mean ± s.d. *P* values of Str or Ctx were determined using Two-tailed Student’s *t*-test. ns, *P* > 0.05; ***P = 0.0041 in **h**. For gel source data, see Supplementary Fig. 1. Source Data

We next examined the origin of the repeat length-dependent increase in m^1^A level in CAG repeat RNA. We tested whether expression of CAG repeat RNA modulates the expression levels of the aforementioned m^1^A writer and eraser—TRMT61A and ALKBH3. respectively. We found no apparent change in expression level of TRMT61A protein in HEK293T cells or mouse tissues expressing different lengths of CAG repeat RNA (Extended Data Fig. 1a–c). By contrast, the levels of ALKBH3 mRNA and protein were diminished in HEK293T cells expressing (CAG)_22_ and (CAG)_38_, with a more pronounced diminution being observed for the latter (Extended Data Fig. 1d–f). In addition, we found that ALKBH3 protein was expressed at a lower level in striatal tissues of Q140 mice than Q7 mice; the expression level of this protein, however, was similar in the cortex tissues of Q140 and Q7 mice (Extended Data Fig. 1g–j). This result is in agreement with the levels of m^1^A detected in the corresponding cells and tissues. Together, these results support that diminished expression of ALKBH3 contributes to elevated frequencies of m^1^A in CAG repeat RNA.

## m^1^A results in neurodegeneration in vivo

We next investigated whether m^1^A in CAG repeat expansion mRNA contributes to neurodegeneration in vivo. We first examined the role of m^1^A in modulating neurodegeneration in *C. elegans* with pan-neuronal expression of 67 repeats of CAG—that is, P_F25B3.3_::Q67::CFP, where CFP is fused to the C-terminus of Q67^32^. On this genetic background, we generated transgenic nematodes expressing BFP-tagged human ALKBH3. In line with previous observations^32^, Q67 worms exhibited pronounced degeneration of the neuron network, as manifested by the loss of neuronal cell bodies and expansion of gaps in neurites of the ventral and dorsal nerve cords (Fig. 1d,f). Expression of wild-type human ALKBH3 (ALKBH3-WT), but not its catalytically inactive mutant (ALKBH3-Mut), diminished the length of the gaps and improved the continuity of dorsal and ventral nerve cords (Fig. 1d,f). Similarly, genetic depletion of *W02A11.1* alleviated neurodegeneration in *C. elegans* (Fig. 1e,f).

**Extended Data Fig. 2:**
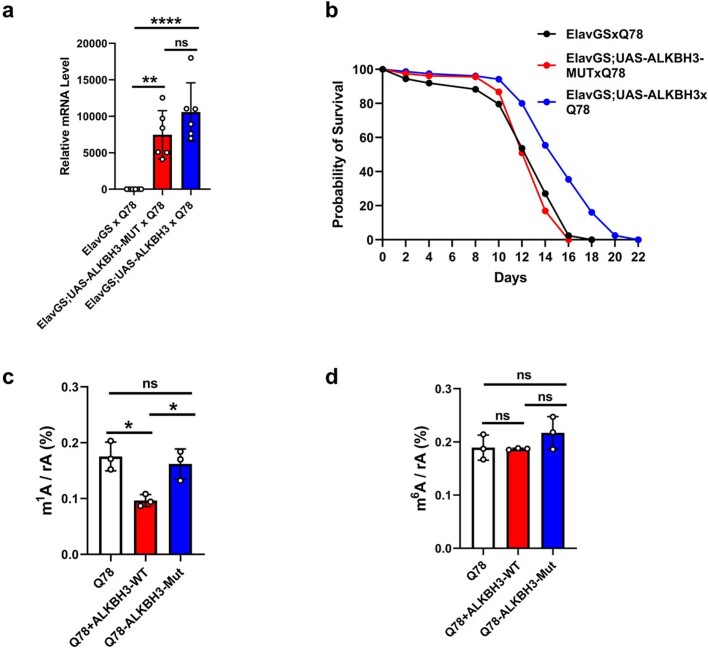
ALKBH3-WT, but not ALKBH3-Mut extends the lifespan of *Drosophila* expressing expanded Q78 CAG repeats, and reduces the ratios of m^1^A/rA, but not m^6^A/rA in CAG repeat RNA. **a**, The mRNA level of wild-type human ALKBH3 (ALKBH3-WT) or its catalytically inactive mutant (ALKBH3-Mut) in Q78 *Drosophila* was measured by RT-qPCR (n = 6 biologically independent samples, 15 heads per sample). Data are mean ± s.d. *P* values were determined using one-way ANOVA with Tukey’s multiple comparisons test. ns, *P* > 0.05; **P = 0.0016; ****P < 0.0001; **b**, *Drosophila* lifespan was evaluated with co-expression of wild-type human ALKBH3 or its catalytically inactive mutant in Q78 *Drosophila* (n = 150 animals per survival curve). **c**-**d**, The levels of m^1^A (**c**) and m^6^A (**d**) in Q78 flies with expression of wild-type human or its catalytically inactive mutant. Data are mean ± s.d., and represent three biologically independent samples (30 heads per sample). Ten-day old male flies on RU486 food were used for the experiments. *P* values were determined using one-way ANOVA with Tukey’s multiple comparisons test. ns, *P* > 0.05; *P = 0.012 between Q78 and Q78 + ALKBH3-WT, and *P = 0.027 between Q78 + ALKBH3-WT and Q78 + ALKBH3-Mut in **c**. Source Data

We also observed an extension of the lifespan in Q78 *Drosophila* with neuronal expression ALKBH3-WT, but not in those expressing ALKBH3-Mut (Extended Data Fig. 2a,b), indicating mitigated neurotoxicity. Our results showed an attenuated level of m^1^A in CAG repeat RNA isolated from Q78 *Drosophila* expressing wild-type human ALKBH3 relative to those expressing its catalytically inactive mutant, with no apparent difference being detected for m^6^A level (Extended Data Fig. 2c,d). These data indicate that m^1^A contributes to neurotoxicity of CAG repeat RNA in *Drosophila*.

## TDP-43 is an m^1^A reader protein

Previously, stable isotope labelling by amino acids in cell culture (SILAC)-based affinity screening led to the identification of several candidate m^1^A-binding proteins, including TDP-43, YTHDF1-3 and DDX56, with TDP-43 exhibiting the highest SILAC protein ratio for m^1^A- over rA-containing probe in proteomic samples prepared from HeLa cells^33^. The mass spectrometry results for QSQDEPLR, a tryptic peptide derived from TDP-43, are shown in Supplementary Fig. 9.

**Extended Data Fig. 3:**
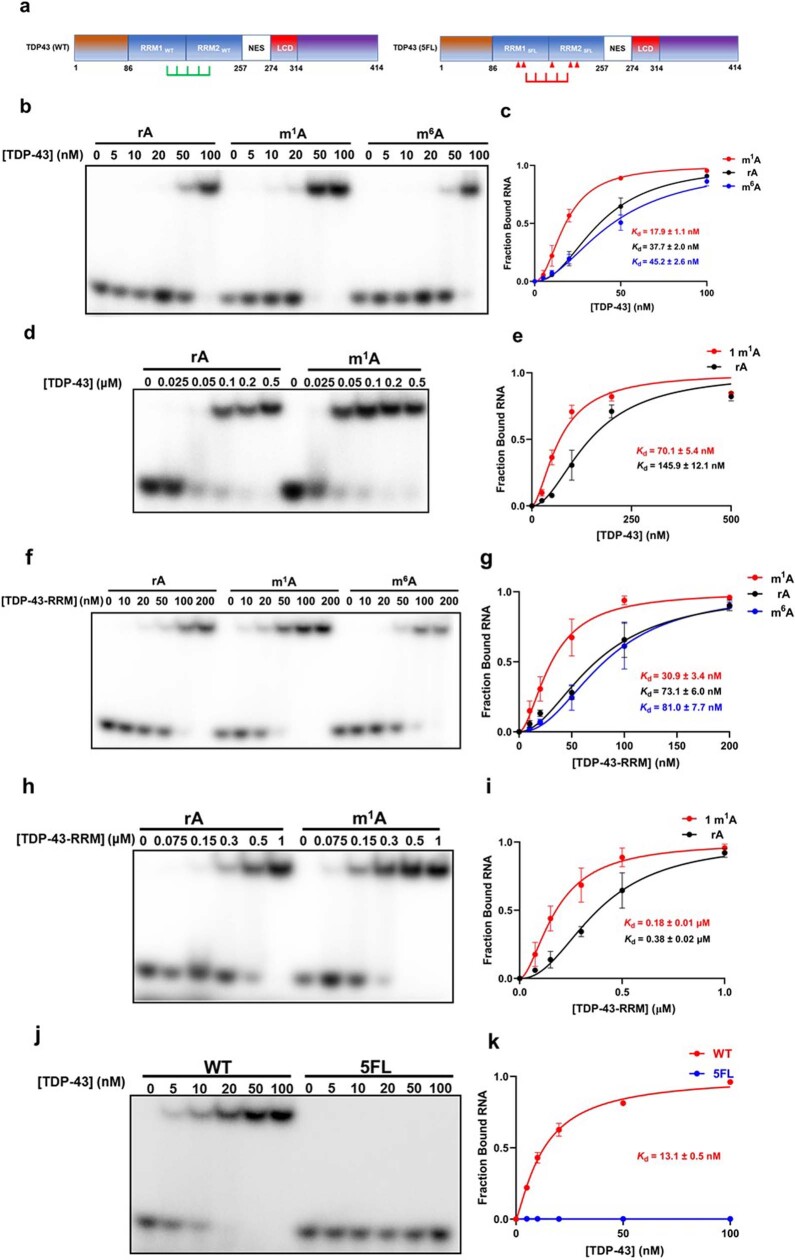
Electrophoretic mobility shift assay (EMSA) for determining the binding affinities of TDP-43 with rA-, m^1^A- and m^6^A-carrying CAG repeat RNA. **a**, Construct designs for TDP-43 vectors containing functional (WT) or RNA-binding-defective (5FL) RRMs were used for EMSA. **b**-**i**, EMSA for measuring the binding affinities of full-length TDP-43 (**b**-**e**) and TDP-43-RRM (**f**-**i**) with rA-, m^1^A- and m^6^A-carrying RNA probes (5′- CCGUUCCGCCCXGGCCGCGCCCAGCUGGAAUGCA-3′, where ‘X’ is rA, m^1^A or m^6^A) (**b**-**c**, **f**-**g**) or with rA-, and m^1^A-carrying CAG repeat RNA probes (5′-CAGCAGCAGCXGCAGCAGCAG-3′, where ‘X’ is rA or m^1^A) (**d**-**e**, **h**-**i**). Protein concentrations ranged from 0 to 1000 nM. Data are mean ± s.e.m., and represent three biologically independent experiments. Shown in (**j**) and (**k**) are the gel image and quantification results for the *K*_d_ values for wild-type TDP-43 and its RRM mutant (TDP-43-5FL) in binding with (CAG)_7_-1 m^1^A. For gel source data, see Supplementary Fig. 1. Source Data

We examined whether TDP-43 can bind directly to m^1^A-containing RNA. Electrophoretic mobility shift assay (EMSA) showed that recombinant TDP-43 binds more strongly to the m^1^A-carrying RNA substrate used in the quantitative proteomic experiment than its unmethylated or m^6^A-containing counterpart, with the dissociation constants (*K*_d_ values) of 18, 38 and 46 nM, respectively (Extended Data Fig. 3b,c). Moreover, we found that recombinant TDP-43 exhibited stronger binding to an m^1^A-carrying CAG repeat RNA than the corresponding unmodified RNA, with *K*_d_ values of 70 and 146 nM, respectively (Extended Data Fig. 3d,e).

TDP-43 contains two RRMs, in which highly conserved aromatic amino acid residues are necessary for the interaction of the protein with RNA, and mutations of 5 Phe residues to Leu in these domains (Extended Data Fig. 3a) abolish the ability of TDP-43 to bind RNA^16^. We observed similar binding selectivity for a truncated version of TDP-43—which contains only the two RRM domains—towards synthetic RNAs with m^1^A, rA and m^6^A, with *K*_d_ values of 31, 73 and 81 nM, respectively (Extended Data Fig. 3f,g). Additionally, we found that the RRM domain exhibits stronger binding to m^1^A-containing CAG repeat RNA than the corresponding unmodified CAG repeat RNA, with the *K*_d_ values of 0.18 and 0.38 μM, respectively, whereas TDP-43-5FL is incapable of binding m^1^A-containing RNA (Extended Data Fig. 3h–k). The purities of recombinant TDP-43 proteins were confirmed by SDS–PAGE analysis (Supplementary Fig. 10).

We next conducted a cross-linking immunoprecipitation–PCR experiment to determine whether TDP-43 binds preferentially to m^1^A in CAG repeat RNA in cells. Our results showed that the ability of TDP-43 to bind with (CAG)_38_ RNA was significantly attenuated in cells with ectopic expression of wild-type ALKBH3, but not with a catalytically inactive mutant (Supplementary Fig. 11a). We also expressed Flag-tagged TDP-43 in HEK293T cells, immunoprecipitated the protein, and quantified the levels of m^1^A in the mRNA samples isolated from the immunoprecipitates by LC–MS/MS. Our results showed that the level of m^1^A was approximately 2.5-fold higher in the anti-Flag pull-down mRNA samples from cells expressing TDP-43–Flag than those expressing the control empty vector (Supplementary Fig. 11b). In addition, the enrichment of m^1^A-bearing mRNA was significantly diminished in the pull-down sample of the corresponding RRM domain mutant TDP-43-5FL–Flag (Supplementary Fig. 11b). We also found that the m^1^A level in (CAG)_38_ RNA was significantly decreased in HEK293T cells upon ectopic co-expression of wild-type ALKBH3, but not its catalytically inactive mutant H257A^34^ (Supplementary Fig. 11c). Immunoblotting experiments results showed similar levels of expression of wild-type and mutant TDP-43, and of ALKBH3 (Supplementary Fig. 11d,e).

We next isolated CAG repeat RNA using affinity pull-down and monitored the level of TDP-43 protein in the pull-down mixture by immunoblot. Our results showed that TDP-43 was present at a higher level in the pull-down mixture of (CAG)_38_ than that of (CAG)_22_, and the amount of TDP-43 in the pull-down mixture was diminished upon ectopic co-expression of ALKBH3 (Supplementary Fig. 11f,g). By contrast, overexpression of ALKBH1 or FTO, or siRNA-mediated knockdown of METTL3—the catalytic subunit of the major m^6^A methyltransferase complex^35^—did not appreciably alter the binding of TDP-43 to (CAG)_38_ (Supplementary Fig. 11h,i). Moreover, (CAG)_38_ RNA did not pull down TDP-43-5FL (Supplementary Fig. 11j). Together, these results show that TDP-43 interacts directly with CAG repeat RNA, and that this interaction requires the RRM domains of TDP-43 and is markedly enhanced by m^1^A in RNA.

## m^1^A induces aberrant TDP-43 behaviour

Truncation, cytoplasmic redistribution and aggregation of TDP-43 are histopathological hallmarks of ALS and FTLD^13^, which, along with our observations of the interaction between this protein and m^1^A-containing RNA, prompted us to examine the effect of CAG repeat RNA on the cleavage and subcellular distribution of TDP-43.

**Extended Data Fig. 4:**
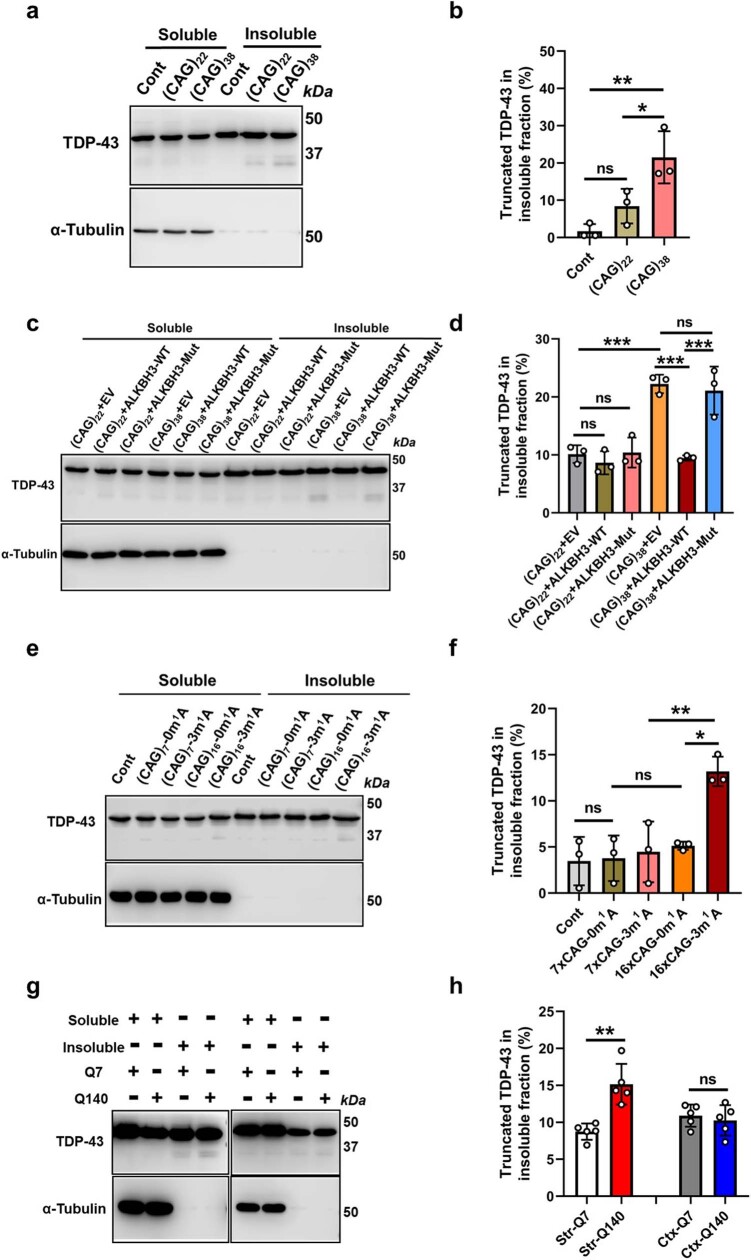
m^1^A in CAG repeat RNA induces truncation of TDP-43 protein. **a**-**f**, Detergent-solubility fractionation and Western blot for endogenous TDP-43 protein in cells with ectopic expression of CAG repeat RNA (**a**, **b**), with or without ectopic expression of ALKBH3-WT or ALKBH3-Mut (**c**, **d**), or with or without transfection of synthetic (CAG)_7_ or (CAG)_16_ RNAs containing zero or three m^1^A (**e**, **f**). Data are mean ± s.d. and represent three biologically independent experiments. *P* values were determined using one-way ANOVA with Tukey’s multiple comparisons test. ns, *P* > 0.05; **P = 0.0066 and *P = 0.041 in **b**; ***P = 0.0004 between (CAG)_22_ + EV and (CAG)_38_ + EV, ***P = 0.0003 between (CAG)_38_ + EV and (CAG)_38_ + ALKBH3-WT, and ***P = 0.0006 between (CAG)_38_ + ALKBH3-WT and (CAG)_38_ + ALKBH3-Mut in **d**; *P = 0.011, and **P = 0.0065 in **f**; **g**-**h**, Quantification results showing the fractions of truncated TDP-43 protein in striatum (n = 5 biologically independent samples) and cortex (n = 5 biologically independent samples) tissues of mice. Data are mean ± s.d. *P* values were determined using Two-tailed Student’s *t*-test. ns, *P* > 0.05; **P = 0.0013. The loading control was detected in a separate gel in parallel for **a**, **c**, **e** and **g**. For gel source data, see Supplementary Fig. 1. Source Data

We were able to detect truncated endogenous TDP-43 in a detergent-insoluble fraction isolated from cells with ectopic expression of (CAG)_22_ and (CAG)_38_ RNA, with (CAG)_38_ RNA being more effective in eliciting truncated TDP-43 protein (Extended Data Fig. 4a,b). Furthermore, ectopic expression of ALKBH3, but not its catalytically inactive mutant, led to diminished levels of truncated TDP-43 in detergent-insoluble fraction isolated from cells expressing (CAG)_38_ RNA (Extended Data Fig. 4c,d). To substantiate the role of m^1^A in CAG repeat RNA in inducing truncated TDP-43 in cells, we used synthetic (CAG)_7_ and (CAG)_16_ RNA carrying zero or three m^1^A residues. Our results showed more truncated TDP-43 in cells transfected with (CAG)_16_-3m^1^A RNA than with the corresponding unmodified RNA, though no apparent difference was detected for transfections with (CAG)_7_-0m^1^A and (CAG)_7_-3m^1^A RNAs (Extended Data Fig. 4e,f). Similarly, we detected a significantly higher level of truncated TDP-43 in the striatal tissues of mice expressing Q140 RNA than those expressing Q7 RNA, but found no such difference in the cortical tissues (Extended Data Fig. 4g,h). These observations are in keeping with the m^1^A levels detected in CAG repeat RNAs isolated from the two types of mouse tissues (Fig. 1c), suggesting that m^1^A in CAG repeat RNA stimulates the generation of truncated TDP-43 protein.

**Fig. 2:**
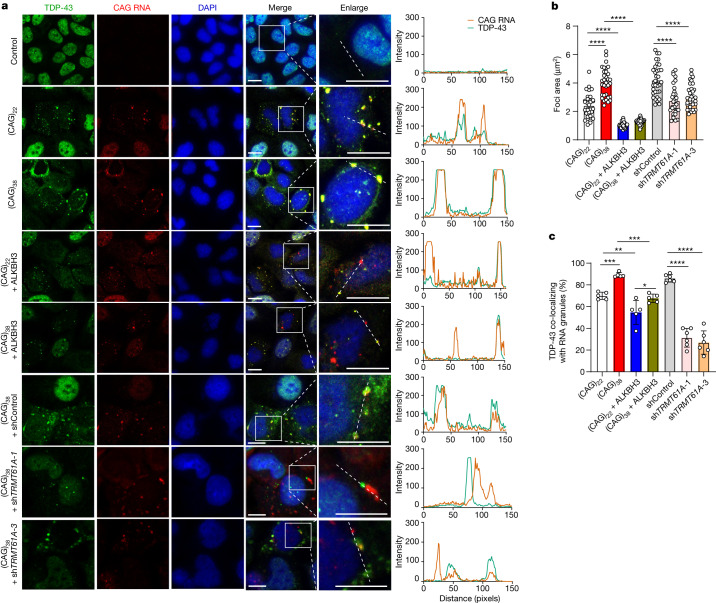
m^1^A induces cytoplasmic mis-localization and aggregation of endogenous TDP-43. **a**, Representative images of U2OS cells expressing CAG repeat RNA along with ectopic expression of ALKBH3 or shRNA-mediated knockdown of TRMT61A. Scale bars, 10 μm. **b**, Quantification of area of TDP-43 foci in **a**. (CAG)_22_: *n* = 31; (CAG)_38_: *n* = 33; (CAG)_22_ + ALKBH3: *n* = 32; (CAG)_38_ + ALKBH3: *n* = 30; shControl: *n* = 33; sh*TRMT61A-1*: *n* = 31; sh*TRMT61A-3*: *n* = 32. **c**, Percentage of TDP-43 co-localized with CAG repeat RNAs. Images are representative of five or six independent frames for each condition. *n* = 5 for (CAG)_22_, (CAG)_38_, (CAG)_22_ + ALKBH3 and (CAG)_38_ + ALKBH3; *n* = 6 for shControl, sh*TRMT61A-1* and sh*TRMT61A-3*. Data are mean ± s.d. and represent three biologically independent experiments. *P* values were determined using one-way ANOVA with Tukey’s multiple comparisons test. *P = 0.020, **P = 0.0059 and ***P = 0.0009 between (CAG)_22_ and (CAG)_38_; ***P = 0.0003 between (CAG)_38_ and (CAG)_38_ + ALKBH3. Source Data

**Extended Data Fig. 5:**
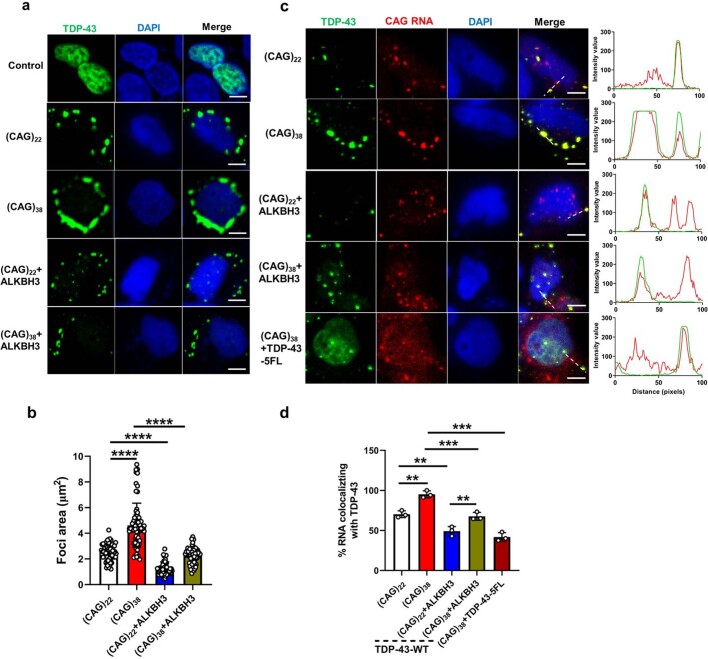
m^1^A triggers cytoplasmic mis-localization and aggregation of ectopically expressed EGFP-TDP-43. **a**, Representative images of U2OS cells co-expressing EGFP-TDP-43 and CAG repeat RNAs with or without ALKBH3 overexpression. **b**, Quantification results for the areas of EGFP-TDP-43 foci. (CAG)_22_: n = 69; (CAG)_38_: n = 69; (CAG)_22_ + ALKBH3: n = 68; (CAG)_38_ + ALKBH3: n = 66. Data are mean ± s.d., and represent three biologically independent experiments. *P* values were determined using one-way ANOVA with Tukey’s multiple comparisons test. ****P < 0.0001. **c**, RNA FISH for assessing the co-localization of EGFP-TDP-43 with CAG repeat RNAs. **d**, Percentages of CAG repeat RNAs co-localized with EGFP-TDP-43. Data are mean ± s.d. and represent three biologically independent experiments. *P* values were determined using one-way ANOVA with Tukey’s multiple comparisons test. **P = 0.0012 between (CAG)_22_ and (CAG)_38_, **P = 0.0033 between (CAG)_22_ and (CAG)_22_ + ALKBH3, **P = 0.0074 between (CAG)_22_ + ALKBH3 and (CAG)_38_ + ALKBH3; ***P = 0.0006 between (CAG)_38_ and (CAG)_38_ + ALKBH3, and ***P = 0.0002 between (CAG)_38_ and (CAG)_38_ + TDP-43-5FL. Scale bar, 10 μm. Source Data

We next examined how expression of CAG repeat RNA modulates the intracellular distribution of TDP-43 protein. Immunostaining for endogenous TDP-43 in U2OS cells showed that whereas TDP-43 is distributed primarily in the nucleus in control cells, ectopic expression of (CAG)_22_ or (CAG)_38_ resulted in cytoplasmic redistribution of TDP-43 (Fig. 2a). In addition, endogenous TDP-43 displayed larger foci in cells expressing (CAG)_38_ than in those expressing (CAG)_22_, where the foci size was decreased upon ectopic expression of ALKBH3 (Fig. 2a,b) or upon genetic depletion of TRMT61A (Fig. 2a,b). Consistent with the results of intracellular distribution of endogenous TDP-43, we observed cytoplasmic mis-localization and aggregation of ectopically expressed TDP-43 in cells expressing (CAG)_38_, where the extent of TDP-43 aggregation was more pronounced than that observed for the endogenous protein (Extended Data Fig. 5a,b).

Next we assessed whether TDP-43 co-localized with CAG repeat RNAs in cells. We combined RNA fluorescence in situ hybridization (FISH) and immunofluorescence assays to monitor subcellular localizations of CAG repeat RNA and TDP-43 protein, respectively. The results showed more extensive co-localization between endogenous TDP-43 and (CAG)_38_ RNA than with (CAG)_22_ RNA (Fig. 2a,c). Additionally, the percentage of this co-localization was significantly attenuated upon ectopic expression of ALKBH3 (Fig. 2a,c) or upon shRNA-mediated knockdown of TRMT61A (Fig. 2a,c). Similar observations were made for ectopically expressed GFP–TDP-43 upon ectopic expression of ALKBH3 (Extended Data Fig. 5c,d); however, ectopically expressed GFP–TDP-43-5FL exhibited impaired co-localization with CAG repeat RNA (Extended Data Fig. 5c,d). This is in keeping with the inability of TDP-43-5FL to bind with CAG repeat RNA (Extended Data Fig. 3j,k).

Together, these results substantiate that CAG repeat RNA binds to TDP-43 in cells, which is driven by m^1^A in the repeat RNA and requires the functional RRM domains of TDP-43. Additionally, CAG repeat RNAs promote the cytoplasmic redistribution and truncation of TDP-43, which again require m^1^A in CAG repeat RNA and its interaction with TDP-43.

## m^1^A drives TDP-43 to stress granules

**Fig. 3:**
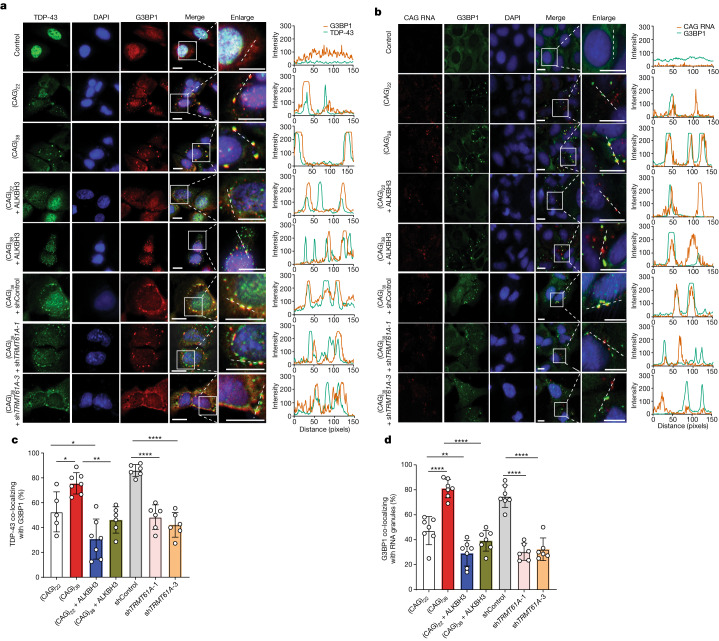
m^1^A enhances the ability of endogenous TDP-43 protein to partition into stress granules. **a**, CAG repeat RNA-mediated localization of endogenous TDP-43 into stress granules in U2OS cells with ectopic expression of ALKBH3 or knockdown of TRMT61A. **b**, FISH and immunofluorescence microscopy were performed to assess the co-localization between G3BP1 and CAG repeat RNAs in U2OS cells with ectopic expression of ALKBH3 or knockdown of TRMT61A. **c**, Percentages of TDP-43 co-localized with G3BP1. Images are representative of 5 to 7 independent frames for each condition: *n* = 5 for (CAG)_22_; *n* = 7 for (CAG)_38_ and (CAG)_22_ + ALKBH3; *n* = 6 for (CAG)_38_ + ALKBH3, shControl, sh*TRMT61A-1* and sh*TRMT61A-3*. **d**, Percentages of G3BP1 co-localized with CAG repeat RNAs. Images are representative of 6 or 7 independent frames for each condition: *n* = 7 for (CAG)_22_, (CAG)_38_, (CAG)_22_ + ALKBH3, (CAG)_38_ + ALKBH3 and shControl; *n* = 6 for sh*TRMT61A-1* and sh*TRMT61A-3*. Data are mean ± s.d. and represent three biologically independent experiments. *P* values were determined using one-way ANOVA with Tukey’s multiple comparisons test. **c**, *P = 0.033 between (CAG)_22_ and (CAG)_38;_ *P = 0.043 between (CAG)_22_ and (CAG)_22_ + ALKBH3; **P = 0.0030. **d**, **P = 0.0072. Scale bars, 10 μm. Source Data

Stress granules are phase-separated compartments in the cytosol^18,36^; we next examined whether CAG repeat RNA and endogenous TDP-43 are localized to stress granules. We observed cytoplasmic foci for endogenous G3BP1 and its co-localization with endogenous TDP-43 in U2OS cells upon expression of (CAG)_22_ and (CAG)_38_, with the extent of co-localization being more pronounced for cells expressing (CAG)_38_ than (CAG)_22_ (Fig. 3a,c). Furthermore, endogenous TDP-43 exhibited diminished co-localization with stress granules upon overexpression of ALKBH3 or genetic depletion of TRMT61A (Fig. 3a,c).

We next determined whether m^1^A-marked RNA was also sequestered into stress granules. Our RNA-FISH and immunofluorescence assay results showed that endogenous G3BP1 granules were co-localized with (CAG)_38_ RNA (Fig. 3b,d). Moreover, the extent of this co-localization was substantially attenuated in cells upon ectopic expression of ALKBH3 or upon shRNA-mediated knockdown of TRMT61A (Fig. 3b,d). Together, these results demonstrated that expression of RNA with expanded CAG repeat is capable of inducing stress granules in cells without heat shock or arsenite treatment. In addition, m^1^A in CAG repeat RNA promotes the sequestration of TDP-43 into stress granules.

**Fig. 4:**
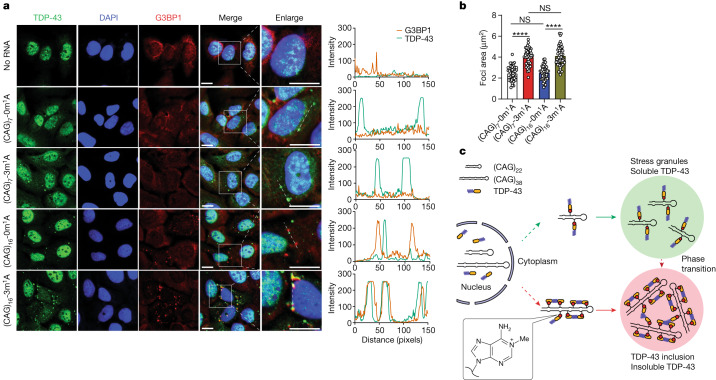
Synthetic m^1^A-containing CAG repeat RNA triggers cytoplasmic redistribution of endogenous TDP-43 and its co-localization with stress granules, and a proposed model illustrating a role of m^1^A in CAG repeat length-dependent modulation of biophysical properties of TDP-43. **a**, Representative images showing the localization of TDP-43 and G3BP1 in U2OS cells with or without transfection with synthetic CAG repeat RNAs containing zero or three m^1^A. Scale bars, 10 μm. **b**, Sizes of TDP-43 foci induced by synthetic CAG repeat RNA containing zero or three m^1^A. *n* = 45 for (CAG)_7_-0m^1^A and (CAG)_7_-3m^1^A; *n* = 46 for (CAG)_16_-0m^1^A and (CAG)_16_-3m^1^A. Data are mean ± s.d. and represent three biological replicates. *P* values were determined using one-way ANOVA with Tukey’s multiple comparisons test. **c**, A model illustrating expanded CAG repeat RNA-induced aberrant phase separation and cytoplasmic redistribution of TDP-43. TDP-43 bound to (CAG)_22_ RNA maintains liquid-like state and is soluble. (CAG)_38_ RNA triggers aberrant phase transition of TDP-43 into insoluble inclusions, in which m^1^A modification in CAG repeat RNA has an important role in stimulating the liquid-to-gel-like transition of TDP-43 by increasing the local concentration of the protein, thereby promoting its aggregation through its LCD. Source Data

To substantiate the role of m^1^A in CAG repeat RNA in influencing the subcellular redistribution and biophysical properties of TDP-43, we used synthetic (CAG)_7_ and (CAG)_16_ RNA carrying zero or three m^1^A residues. Transfection of cells with the synthetic CAG repeat RNAs led to cytoplasmic redistribution of TDP-43 in a sub-population of cells, and transfection with (CAG)_7_-3m^1^A or (CAG)_16_-3m^1^A RNA conferred larger TDP-43 foci in the cytosol than with the same amount of the corresponding unmodified RNAs (Fig. 4a,b). The observation of cytoplasmic redistribution of TDP-43 in cells transfected with the unmodified CAG repeat RNAs may be owing to the modification of some of the rA in the repeat RNAs to m^1^A in cells after transfection.

We also observed co-localization between endogenous TDP-43 and G3BP1 after transfecting cells with the synthetic CAG repeat RNAs, with a higher degree of co-localization being observed in cells transfected with (CAG)_16_-3m^1^A than in those with the corresponding unmodified RNA (Fig. 4a). Together, these results support the proposition that m^1^A is a crucial molecular determinant in inducing cytoplasmic mis-localization of TDP-43 and its co-localization with stress granules.

## m^1^A alters phase separation of TDP-43

**Extended Data Fig. 6:**
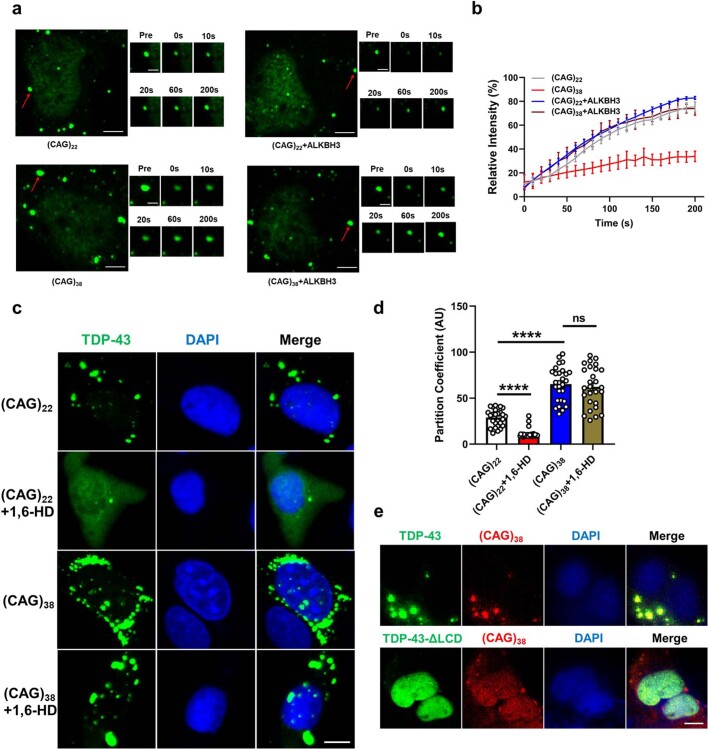
(CAG)_38_ RNA triggers phase transition of TDP-43 protein in cells. **a**, FRAP of EGFP-TDP-43 droplets in U2OS cells at 24 h following transfection. Scale bar, 5 μm. Higher magnification is shown in the right panel at different time points following photobleaching at 0 s (scale bar, 2 μm). **b**, Mean fluorescence intensity of EGFP-TDP-43 in the fully bleached area over time. Data are mean ± s.e.m. from the recovery curves obtained from three biologically independent experiments. **c**, Representative images of U2OS cells expressing (CAG)_22_ RNA with or without treatment with 6% 1,6-hexanediol (1,6-HD) for 1 min. Scale bar, 10 μm. **d**, Partition coefficients of TDP-43 protein in (CAG)_22_- or (CAG)_38_-expressing cells with or without treatment with 6% 1,6-HD. The partition coefficient was calculated based on the fraction of EGFP-TDP-43 signal in the droplets over EGFP-TDP-43 signal in immediately adjacent region. n = 27 foci of EGFP-TDP-43 for each group with three biologically independent experiments. Data are mean ± s.e.m. *P* values were determined using Two-tailed Student’s *t*-test. ns, *P* > 0.05; ****P < 0.0001. **e**, Phase separation of EGFP-TDP-43 and EGFP-TDP-43-ΔLCD proteins in U2OS cells with or without ectopic expression of (CAG)_38_ mRNA. The experiments were repeated independently three times with similar results. Scale bar, 10 μm. Source Data

We next explored whether TDP-43 exhibits phase-separated liquid-like properties in cells and how these properties may be modulated by the interaction of TDP-43 with m^1^A in CAG repeat RNA. We first examined the physical properties of GFP–TDP-43 in cells expressing RNA with expanded CAG repeats. Our results showed that after photobleaching, the fluorescence of GFP–TDP-43 was rapidly recovered in cells with ectopic expression of (CAG)_22_ RNA (Extended Data Fig. 6a,b), suggesting that a large fraction of TDP-43 protein is mobile in cells expressing (CAG)_22_ RNA. However, we observed little recovery of GFP–TDP-43 fluorescence after photobleaching in cells expressing (CAG)_38_ RNA, indicating that TDP-43 protein is immobile in these cells (Extended Data Fig. 6a,b). Furthermore, ectopic co-expression of ALKBH3 with (CAG)_38_ restored rapid fluorescence recovery of GFP–TDP-43 after photobleaching (Extended Data Fig. 6a,b).

To further investigate the biophysical properties of TDP-43 granules, we treated cells that ectopically co-expressed TDP-43 and (CAG)_38_ or (CAG)_22_ with 1,6-hexanediol (1,6-HD), which disrupts liquid-like condensates^37^. Upon treatment with 1,6-HD, there were fewer GFP–TDP-43 granules in cells expressing (CAG)_22_ (Extended Data Fig. 6c), again substantiating that TDP-43 is mobile and exhibits liquid-like properties in these cells. The granules in cells expressing (CAG)_38_, however, displayed markedly reduced sensitivity toward 1,6-HD (Extended Data Fig. 6c,d). Moreover, we found that truncated TDP-43 lacking the LCD (TDP-43-ΔLCD), along with (CAG)_38_ RNAs, was located mainly in the nucleus and exhibited a diffused pattern (Extended Data Fig. 6e). Together, this suggests that long CAG repeat RNA expansions facilitate a liquid-to-gel-like transition of TDP-43, which entails m^1^A in the repeat RNA and the LCD of TDP-43.

**Extended Data Fig. 7:**
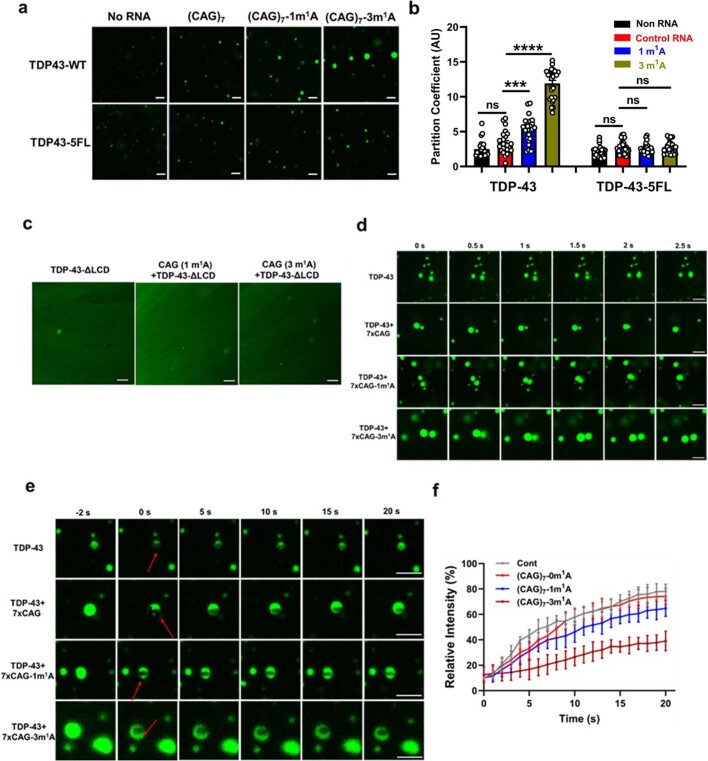
m^1^A modulates liquid-liquid phase separation of TDP-43 protein. **a**, The droplets of EGFP-TDP-43 and EGFP-TDP-43-5FL were detected in the presence of synthetic (CAG)_7_ RNAs (50 ng) containing 0, 1 and 3 m^1^A residues, or without RNA. **b**, Partition coefficients of wild-type and mutant TDP-43 proteins in the presence or absence of m^1^A-containing CAG repeat RNA. 24 or 25 TDP-43-EGFP droplets observed in three biologically independent experiments for each group were quantified. n = 24 for TDP-43-WT + No RNA and TDP-43-WT + 3m^1^A; n = 25 for TDP-43-WT + Control RNA, TDP-43-WT + 1m^1^A,TDP-43-5FL + No RNA, TDP-43-5FL + Control RNA, TDP-43-5FL + 1m^1^A, and TDP-43-5FL + 3m^1^A. Data are mean ± s.e.m. *P* values were determined using one-way ANOVA with Tukey’s multiple comparisons test. ns, *P* > 0.05; ***P = 0.0009; ****P < 0.0001. **c**, The droplets of TDP-43-ΔLCD were detected with CAG repeats RNAs (50 ng) or without RNA. Scale bar, 5 μm. **d**, TDP-43-EGFP (50 μM) was imaged using confocal microscopy. The fusions of TDP-43-EGFP droplets are also shown in Supplementary Videos 1–4. Scale bar, 5 μm. The experiments shown in **c** and **d** were repeated at least three times with similar results. **e**, Representative examples of FRAP analyses of in vitro phase-separated TDP-43 droplets in the presence of synthetic (CAG)_7_ RNAs containing 0, 1 and 3 m^1^A residues, or without RNA. FRAP are also shown in Supplementary Videos 5–8. **f**, FRAP curves displaying the relative TDP-43 intensity of phase-separated droplets in the presence of (CAG)_7_ RNAs containing 0, 1 and 3 m^1^A residues, or without RNA. The data represented mean ± s.e.m. from the recovery curves obtained from three biologically independent experiments. Scale bar, 2 μm. Source Data

We next tested whether m^1^A regulates phase separation of TDP-43 protein in vitro using synthetic (CAG)_7_ RNA containing 0, 1 or 3 m^1^A residues. Although the unmodified (CAG)_7_ RNA did not alter the phase separation of TDP-43 (Extended Data Fig. 7a), the corresponding RNAs carrying one or three m^1^A residues triggerred the formation of large droplets and increased the partition coefficient of TDP-43 in the droplets (Extended Data Fig. 7a,b). Unlike wild-type TDP-43, the phase separation of TDP-43-5FL was not influenced by the presence of CAG repeat RNAs (Extended Data Fig. 7a,b). Moreover, TDP-43-ΔLCD did not form droplets in vitro; this effect was independent of the presence of CAG repeat RNAs (Extended Data Fig. 7c).

We also examined whether synthetic m^1^A-containing RNAs influence the biophysical properties of the TDP-43 droplets in vitro. We observed that the protein droplets fused to form larger droplets within 2.5 s in the presence of (CAG)_7_ RNA with 0 or 1 m^1^A, indicating that TDP-43 protein exhibits LLPS (Extended Data Fig. 7d and Supplementary Videos 1–3). The TDP-43 droplets, however, coalesced more slowly upon incubation with (CAG)_7_-3m^1^A (Extended Data Fig. 7d and Supplementary Video 4). Photobleaching experiments revealed that TDP-43 assembles into gel-like aggregates upon incubation with (CAG)_7_-3m^1^A (Extended Data Fig. 7e,f and Supplementary Videos 5–8). These in vitro results corroborate our findings from cellular experiments and demonstrate that m^1^A in CAG repeat RNA perturbs the phase separation of TDP-43 protein by rendering the protein much less mobile.

Our findings parallel a recent observation that multivalent m^6^A-containing RNAs can enhance the phase separation potential of YTHDF proteins in vitro and in cells^38,39^. Notably, our results revealed a repeat length-dependent accumulation of m^1^A in CAG repeat RNA, and a novel pathological function of m^1^A—that is, in binding with TDP-43 and eliciting its aberrant biochemical and biophysical properties that recapitulate the observations made for the protein in many neurological diseases (Fig. 4c). It is important to note that neurotoxicity observed in CAG repeat expansion disorders may also arise from polyglutamine-containing proteins translated from the repeat-bearing RNAs^3,4^, and the ensuing perturbation of the ubiquitin–proteasome system^11^. Thus, multiple mechanisms probably contribute to neurodegenerative diseases caused by nucleotide repeat expansions.

In sum, our work shifts the current paradigm of TDP-43 proteinopathy and suggests new ways to treat neurological diseases. Our identification of the methyltransferase (TRMT61A) and demethylase (ALKBH3) for m^1^A in CAG repeat RNA provide a strong foundation for such future efforts.

## Methods

### Cell culture and shRNA knockdown

HEK293T cells and U2OS cells were obtained from ATCC. The cells were maintained in DMEM (11995-065, Gibco) with 10% FBS, 100 U ml^−1^ penicillin and 100 μg ml^−1^ streptomycin under standard cell culture conditions (37 °C, 5.0% CO_2_). The cell lines were authenticated by ATCC using short tandem repeat (STR) analysis as described in 2012 in ANSI Standard (ASN-0002) Authentication of Human Cell Lines. The cells were confirmed to be free of mycoplasma contamination by using LookOut Mycoplasma PCR Detection Kit (MP0035, Sigma-Aldrich).

shRNAs against *TRMT61A*, *TRMT61B* and *TRMT10C* were designed and their sequences are listed in Supplementary Table 3. All shRNAs and control non-targeting shRNAs were cloned into the AgeI/EcoRI site of the pLKO.1 vector (Addgene, plasmid #10878) and confirmed by Sanger sequencing. Cells were transfected with pLKO.1/puro-shRNAs together with pLTR-G (Addgene plasmid #17532) envelope plasmid and pCMV-dR8.2 dvpr (Addgene plasmid #8455) package plasmid using PolyFect transfection reagent (QIAGEN). Viral particles were collected 48 h later and filtered through a 0.45-μm sterile filter. After that, cells were transfected with lentiviral constructs expressing shRNA for 24 h, and selected with puromycin for 7 days.

### Extraction of CAG repeat mRNA and LC–MS/MS measurements

Total RNA was extracted from HEK293T cells using TRI reagent (Sigma). The RNA sample (~20 μg) was subsequently incubated with 200 μM of 5′-biotinylated 5×CTG oligodeoxyribonucleotides in a 2-volume hybridization buffer (50 mM Tris-HCl, pH 7.0, 750 mM NaCl, 1 mM EDTA, 1% SDS and 15% formamide) at room temperature for 4 h with gentle mixing. To the resulting mixture were then added streptavidin-conjugated agarose beads (Thermo Scientific) and the suspension was incubated at 4 °C for 2 h. After the incubation, the oligodeoxyribonucleotide-bound beads were washed for four times with 2× SSC buffer (0.30 M NaCl, 15 mM sodium citrate, pH 7.0) and the beads was resuspended in RNase-free water for digestion.

For the cellular pull-down experiment, HEK293T cells were transfected with Flag-tagged TDP-43 or 5FL mutant plasmid. After a 24-h incubation, the cells were washed with PBS and lysed in a buffer containing 10 mM HEPES (pH 7.5), 150 mM KCl, 2 mM EDTA, 0.5% IGEPAL CA-630, 0.5 mM DTT, protease inhibitor (Sigma), and 40 units ml^−1^ RNase inhibitor. The supernatant was incubated with anti-Flag M2 beads (Sigma) at 4 °C overnight. The beads were washed for three times with a buffer containing 50 mM HEPES (pH 7.5), 200 mM NaCl, 2 mM EDTA, 0.05% IGEPAL CA-630, and 0.5 mM DTT. The TDP-43-bound RNA was extracted from the beads using TRI reagent, and mRNA was isolated and purified by using PolyATtract mRNA Isolation System IV (Promega) according to the manufacturer’s instructions.

The above affinity-purified RNA (400 ng) was digested with 1 unit of nuclease P1 in 25 μl buffer containing 25 mM NaCl and 2.5 mM ZnCl_2_. The mixture was then incubated at 37 °C for 2 h, and to the mixture were added 0.5 unit of Antarctic phosphatase and 3 μl of 1.0 M NH_4_HCO_3_. After incubation at 37 °C for an additional 2 h, the digestion mixture was dried and reconstituted in 100 μl ddH_2_O. [^13^C_5_]-adenosine, [D_3_]-m^6^A, and [D_3_]-m^1^A were used as internal standards for the quantifications of rA, m^6^A, and m^1^A, respectively. In parallel, total RNA (400 ng as input control RNA) was digested in the same way. The enzymes in the digestion mixture were removed by extraction using chloroform:isoamyl alcohol (24:1), and salt in the samples was removed by acetonitrile precipitation. The resulting supernatant was dried, and the dried residues were reconstituted in 10 μl ddH_2_O and injected for LC–MS/MS analysis on a TSQ Altis triple-quadrupole mass spectrometer (Thermo). For m^1^A and m^6^A quantifications, the pre-column and analytical column were packed with porous graphitic carbon and Zorbax SB-C18 stationary phase materials, respectively, where a gradient of 0–15% B in 10 min, 15–95% B in 30 min, and 95% B in 10 min was used. The mass spectrometer was operated in the multiple-reaction monitoring (MRM) mode, where the loss of a ribose from the [M + H]^+^ ions of rA, m^6^A, m^1^A and their stable isotope-labelled counterparts were monitored.

RNase H was used to remove the 5′ and 3′ flanking RNA sequences of the (CAG)_38_ repeat. In particular, the isolated RNA was annealed in the RNase H reaction buffer (New England Biolabs) by heating to 95 °C and cooling slowly to 70 °C. The temperature was held at 70 °C for 10 min to melt nonspecific RNA structures, before cooling slowly to 37 °C. RNase H was added to the mixture, incubated at 37 °C for 2 h, and the reaction was terminated with 0.5 M EDTA. (CAG)_38_ RNA was subsequently isolated by affinity purification, digested with enzymes, and analysed by LC–MS/MS, as described above. Unless specifically noted, the reported levels for m^1^A and m^6^A in CAG repeat RNA were without RNase H treatment.

C57BL/6 mice expressing one wild-type endogenous Htt allele and a second Htt allele with knock-in of human *mHTT* exon 1 containing 140 CAG repeats were described previously^31,40^. All mice were maintained and bred under standard conditions consistent with National Institutes of Health guidelines and approved by the University of California, Los Angeles Institutional Animal Care and Use Committee. The cages were maintained on a 12:12 light:dark cycle, with food and water ad libitum. Striatum and cortex tissues were harvested from wild-type and heterozygous HD knock-in Q140 mice at six months of age and immediately frozen on dry ice, where tissues from both male and female mice were used. Q19 (AM49, rmls172), Q40 (AM101, rmls110) and Q67 worm strains (AM44, rmls190) were purchased from *Caenorhabditis* Genetics Center (CGC). *Drosophila* expressing a SCA3 transgene with 78 repeats of CAG (UAS-MJDtrQ78) and those with a short (UAS-HA-MJDtrQ27 line c19.1) CAG repeat (Bloomington *Drosophila* Stock Center line 8149) have been described^6,41^, where the transgene was expressed in neurons using ElavGal4 (Bloomington line 458). Coding sequences of wild-type and catalytically inactive mutant human *ALKBH3* were subcloned into a pUAST transformation vector, and transgenic fly lines were subsequently generated (chrIII, attP2). CAG repeat RNA was isolated from total RNA extracted from mouse striatum and cortex tissues, *Drosophila* heads and *C. elegans*, digested, and analysed in a similar way as described above for cellular RNA.

The levels of rA, m^6^A and m^1^A were quantified by employing calibration curves obtained from pure nucleoside standards analysed under the same instrument conditions (Supplementary Figs. 2 and 3). We also assessed the recovery rate of the analytical workflow in measuring m^1^A and m^6^A in CAG repeat RNA by mixing (CAG)_7_-0m^1^A with (CAG)_7_-1m^1^A and (CAG)_7_-1m^6^A at defined m^1^A/rA and m^6^A/rA molar ratios, and quantified the levels of m^1^A and m^6^A by following the same procedures as described above for CAG repeat RNA isolated from cells and tissues. The recoveries of m^1^A and m^6^A were determined to be approximately 85% and 87%, respectively (Supplementary Fig. 12). Given the relatively high recovery rates, the reported measurement results for m^1^A and m^6^A were without correction for the recovery rates.

### Pull-down of TDP-43 with CAG repeat RNA and western blot analysis

HEK293T cells were transfected with a plasmid encoding Flag–TDP-43 or Flag–TDP-43-5FL together with the indicated CAG repeat plasmid. The cells were harvested at 70–80% confluence, washed with PBS, and lysed in CelLytic M cell lysis reagent (Sigma). The lysates were centrifuged at 13,000 rpm for 10 min at 4 °C. The supernatant was pre-cleared by incubating with streptavidin-conjugated agarose beads (Thermo Scientific) at 4 °C for 1 h, and 20 μl samples were taken as inputs. Biotinylated RNA baits (3 µg) were incubated, at room temperature for 4 h, with pre-cleared cell lysates in a hybridization buffer containing protease and RNase inhibitors. Streptavidin-conjugated agarose beads were then added to the mixture, and the mixture was kept in a shaker at 4 °C for 2 h. The beads were washed extensively and boiled for 10 min, and the supernatant collected. The samples were subsequently resolved by 10% SDS–PAGE and transferred to a PVDF membrane (Durapore membrane filter, 0.45 μm, Millipore). Membranes were blocked in 5% milk at room temperature for 1 h, and then incubated with primary antibodies in 5% BSA at 4 °C overnight. Primary antibodies for the following proteins or epitope were used: TDP-43 (Proteintech, 10782-2-AP, 1:1,000), G3BP1 (Proteintech, 66486-1-lg, 1:1,000), TRMT61A (Thermo Fisher, A305-858A-T, 1:1,000), Flag epitope (Cell Signaling Technology, 14793 S, 1:2,000), ALKBH3 (Cell Signaling Technology, 87620, 1:1,000), GAPDH (Santa Cruz Biotechnology, sc-32233, immunoblot, 1:5,000), and α-tubulin (Santa Cruz Biotechnology, sc-32293, 1:5,000). After washing with PBS containing 0.05% Tween (PBS-T), the membranes were incubated with secondary antibodies at room temperature for 1 h. Secondary antibodies used for western blotting included anti-rabbit IgG (whole molecule)-Peroxidase antibody produced in goat (Sigma, A0545, 1:2500) and m-IgGκ BP-HRP (Santa Cruz Biotechnology, sc-516102, 1:2500). Membranes were then washed with PBS-T and bands were visualized using a LI-COR imaging system and quantified by using ImageJ. All western blots shown are representative results obtained from at least three biological replicates.

### Detergent solubility fractionation assay of TDP-43

Detergent solubility fractionation of TDP-43 was performed as described^42^. In brief, cells were washed once with ice-cold PBS, lysed with CelLytic M cell lysis reagent (Sigma), and incubated on ice for 10 min. Following a brief sonication on ice, lysates were centrifuged for 40 min at 15,000*g* at 4 °C. Supernatants were collected as the detergent-soluble fraction. The pellets were subsequently resuspended in a urea buffer (30 mM Tris pH 7.5, 7 M urea, and protease inhibitor cocktail), briefly sonicated on ice, and lysates were centrifuged at 15,000*g* at room temperature for 1 h, where supernatant was collected as the detergent-insoluble, urea-soluble fraction. Proteins in the detergent-soluble and insoluble fractions were then resolved by SDS–PAGE and analysed by western blot.

### Expression and purification of recombinant proteins

Recombinant TDP-43 proteins were purified by following previously described procedures^43^. For purification of full-length TDP-43–MBP–6×His, TDP-43-5FL–MBP–His, TDP-43-5FL–eGFP–MBP–His and TDP-43-ΔLCD–eGFP–MBP–His proteins, BL21-DE3 *Escherichia coli* cells were cultured at 37 °C to an OD at 600 nm of 0.6–0.9, and induced with 1 mM isopropyl β-d-1-thiogalactopyranoside (IPTG) at 18 °C for 16 h. The cells were subsequently collected, pelleted and resuspended in a binding buffer containing 20 mM Tris-HCl (pH 8.0), 1 M NaCl, 10% (v/v) glycerol, 1 mM DTT, and EDTA-free protease inhibitor cocktail according to the manufacturer’s instructions. The cells were lysed by sonication and centrifuged at 10,000*g* for 20 min. The proteins were purified over pre-packed Dextrin Sepharose High Performance MBPTrap HP (MBPTrap HP columns, GE) and eluted by using the binding buffer containing 10 mM maltose. Proteins were further purified over pre-packed Ni Sepharose High Performance HisTrap HP (GE) and eluted with a buffer containing 50 mM Tris (pH 7.4), 500 mM NaCl and 400 mM imidazole. Protein concentration was determined by Bradford assay (Bio-Rad). The eluted fractions were analysed by using denaturing SDS–PAGE, and the purities of the recombinant proteins were assessed by SDS–PAGE analysis.

### Electrophoretic mobility shift assay

RNA probe, 5′-biotin-CCGUUCCGCCCXGGCCGCGCCCAGCUGGAAUGCA-3′ (where X = m^1^A, A or m^6^A; Supplementary Table 2), was radiolabelled at the 3′ terminus, following previously described procedures^44^. In brief, [5′-^32^P]cytidine-3′,5′-bis(phosphate) (pCp) was prepared by incubating 0.5 μl 0.5 mM cytidine-3′-monophosphate (Cp, Carbosynth) with 1.0 μl 10× T4 PNK buffer (NEB), 1 μl T4 PNK (3′ phosphatase minus, NEB), and 8.25 μl [γ-^32^P]ATP at 37 °C for 1 h. The mixture was incubated at 65 °C for 10 min. RNA probe was 3′-end labelled in a 30-μl buffer containing 3 μl 10× T4 RNA ligase buffer (NEB), 3 μl 10 mM ATP (NEB), 1 μl 100 mM DTT (Promega), 3 μl DMSO (Sigma), 9.1 μl [5′-^32^P]pCp, and 3 μl T4 RNA ligase I (NEB) at 4 °C for 24 h. The probes were then purified by using micro bio-spin P-30 columns (Bio-Rad). In addition, synthetic 21-mer (CAG)_7_ RNA with or without m^1^A (Supplementary Table 2) were labelled with ^32^P on the 5′ termini by following standard procedures and used for EMSA experiments. In brief, the probe (20 fmol) was incubated with increasing concentrations of TDP-43 or its truncated or mutated variants in a binding buffer (10 mM HEPES, pH 8.0, 50 mM KCl, 1 mM EDTA, 5% glycerol, 1 mM DTT, 40 units ml^−1^ RNase inhibitor) at 4 °C for 1 h. The entire 20-µl sample was subjected to electrophoresis on an 8% native polyacrylamide gel and the gel band intensities were quantified using phosphorimager analysis with a Typhoon 9410 Variable Mode Imager. The dissociation constant (*K*_d_) was calculated using Prism software (version 8.0.1).

### Quantification of TDP-43 granule size

For quantification of TDP-43 granule size in U2OS cells expressing RNA with different lengths of CAG repeat, the cells were fixed using methanol and 20–35 granules were analysed per condition. RNA granules were enumerated, and the percentages of granules co-localized with the stress granule marker (G3BP1) were calculated. The intensity and area of co-localized granules were calculated using plot profile tool in Fiji.

### Fluorescence recovery after photobleaching

FRAP experiments were performed using an LSM 880 laser scanning confocal microscope (Zeiss 880 Inverted Airyscan Fast) coupled with a temperature-, humidity- and CO_2_-controlled top-stage incubator for live-cell imaging. Photobleaching was conducted by a 488-nm line from an Argon laser at 50% power. Three regions of interest (ROIs) were defined for these experiments: ROI-1 was the indicated circular region in the droplet; ROI-2 was a circular, un-photobleached region of similar size in the same droplet; and ROI-3 was defined as background and drawn outside of the droplet, where its signal was subtracted from those of ROI-1 and ROI-2. Raw data were processed and plotted using Prism.

### RNA-FISH and immunofluorescence microscopy

U2OS cells expressing the indicated RNA were fixed at 24 h following transfection and permeabilized by incubation for 10 min in methanol containing 10% (v/v) acetic acid. RNA was detected using a Cy3-labelled DNA probe (5′-/5Cy3/CTGCTGCTGCTGCTGCTGCTGCTG-3′). Hybridization and washing buffers were obtained from Biosearch Technologies and used following the manufacturer’s protocol. For immunofluorescence detection of proteins following RNA-FISH, methanol-fixed cells were stained using antibodies against TDP-43 (Proteintech, 10782-2-AP, 1:100) and G3BP1 (Proteintech, 66486-1-lg, 1:100), and Alexa Fluor 488 and 647-labelled secondary antibodies (Invitrogen, A-32731, 1:250, and Invitrogen, A-32728, 1:250, respectively). The samples were subsequently stained with DAPI and imaged using confocal microscopy as described above. The data were analysed using Zen 2 Blue software (version 2.3).

### In vitro phase separation assays

In vitro TDP-43 droplet formation was induced in a buffer containing 20 mM HEPES (pH 7.4), 300 mM KCl, 6 mM MgCl_2_, 0.02% NP-40, and 50% glycerol. Dextran (10%) was also added as a crowding agent to induce phase separation of TDP-43. The droplets on a coverslip were detected using a 100× oil immersion objective by confocal microscopy. After TDP-43 droplet formation following the addition of m^1^A-RNA, partition coefficients were calculated for stably formed TDP-43-containing droplets based on the ratio of intensity of TDP-43 in phase-separated droplets over that located in the immediately adjacent region.

### In vitro methyltransferase assay with TRMT6–TRMT61A

The expression plasmid, pET17b-6×HisTrm6-Trm61, was isolated from a strain provided by J. Finer-Moore^45^ and was modified to contain a 3C protease cleavage sequence C-terminal to the 6×His tag. Recombinant TRMT6–TRMT61A was purified following previously described procedures^45^. For the methyltransferase assay, synthetic (CAG)_16_ RNA was annealed by first heating to 95 °C for 5 min and then cooling to 25 °C. The reactions were performed at room temperature overnight in 20 μl buffer containing 50 mM ammonium acetate, 3 mM MgCl_2_, 50 mM Tris-HCl (pH 8.0), 1 mM DTT, 1 mM *S*-adenosylmethionine, 2.5 μM RNA substrate and 12.5 μM TRMT6–TRMT61A complex. After the reaction, proteins were removed by chloroform extraction, and the RNA was subsequently digested by nuclease P1 and Antarctic phosphatase, and the levels of rA and m^1^A were measured by LC–MS/MS, as described above.

### *C. elegans* strains and maintenance

*C. elegans* strains were maintained at 20 °C on standard nematode growth medium agar plates seeded with *E. coli* OP50 bacteria unless otherwise stated^46^. A list of strains used in this study is provided in Supplementary Table 4.

To generate transgenic *C. elegans* strains (WG291 and WG300; Supplementary Tables 4) expressing human ALKBH3 (hALKBH3) driven by a pan-neuronal snb-1 promoter, we cloned 1.5 kb of DNA upstream of the *snb-1* gene, which is specifically expressed in the somatic nervous system. The cDNA of human *ALKBH3* gene was obtained from HEK293T cells and amplified by PCR. Two introns were inserted into *ALKBH3* to avoid mis-splicing. This *ALKBH3*-WT cDNA was used in site-directed mutagenesis experiments to generate the catalytically inactive mutant of ALKBH3. Blue fluorescence protein (BFP) was fused to the C-terminus of ALKBH3 to monitor the expression of ALKBH3 protein in worms. All fragments with *TBB2* 3′-UTR were ligated by employing PCR walking technique and inserted into pCR2.1-TOPO (Thermo Fisher). The primer sequences are listed in Supplementary Table 1. The plasmid was injected together with the *rol-6 (su1006)* dominant marker plasmid pRF4 into the germline cells of early-adult hermaphrodites, where the injection mixtures contained 50 μg ml^−1^ each of pRF4 and ALKBH3 plasmids^47^. The F_1_ progenies of the transgenic strains generated in this manner carried heritable extrachromosomal multi-copy arrays of both the experimental and marker plasmids and were used for microscopy assessment. The gaps between neurons were quantified using ImageJ.

For RNAi experiments, *W02A11.1* RNAi constructs were obtained from the Vidal RNAi library. Hermaphrodite worms were fed *E. coli* OP50 containing an empty control vector (L4440) or expressing double-stranded RNA when they reached adulthood^48^. Plates for RNAi analysis were prepared by supplementing agar with 100 μg ml^−1^ carbenicillin and 1 mM IPTG after autoclave. Worms were synchronized and grown from hatching on RNAi-feeding plates unless otherwise stated. RNAi efficiency was verified by quantitative PCR with reverse transcription and western blot.

### Statistical analysis

All statistical analyses were performed in GraphPad Prism (version 8.0.1) or Microsoft Excel 2016. The outcomes of all statistical tests including *P* values and number of samples are included in the figure panels or the corresponding figure legends. Significance was defined as any statistical outcome that resulted in a *P* value of less than 0.05, unless otherwise indicated^23,49^.

### Reporting summary

Further information on research design is available in the Nature Portfolio Reporting Summary linked to this article.

## Online content

Any methods, additional references, Nature Portfolio reporting summaries, source data, extended data, supplementary information, acknowledgements, peer review information; details of author contributions and competing interests; and statements of data and code availability are available at 10.1038/s41586-023-06701-5.

### Supplementary information

Supplementary Information Supplementary Tables 1–4 and Supplementary Figs 1–12.

Reporting Summary

Supplementary Video 1 Fusion events of TDP-43–eGFP with or without synthetic (CAG)_7_ RNAs containing 0, 1 or 3 m^1^A.

Supplementary Video 2 Fusion events of TDP-43–eGFP with or without synthetic (CAG)_7_ RNAs containing 0, 1 or 3 m^1^A.

Supplementary Video 3 Fusion events of TDP-43–eGFP with or without synthetic (CAG)_7_ RNAs containing 0, 1 or 3 m^1^A.

Supplementary Video 4 Fusion events of TDP-43–eGFP with or without synthetic (CAG)_7_ RNAs containing 0, 1 or 3 m^1^A.

Supplementary Video 5 Fluorescence recovery after photobleaching (FRAP) of TDP-43–eGFP in the presence or absence of synthetic (CAG)_7_ RNAs containing 0, 1 or 3 m^1^A.

Supplementary Video 6 Fluorescence recovery after photobleaching (FRAP) of TDP-43–eGFP in the presence or absence of synthetic (CAG)_7_ RNAs containing 0, 1 or 3 m^1^A.

Supplementary Video 7 Fluorescence recovery after photobleaching (FRAP) of TDP-43–eGFP in the presence or absence of synthetic (CAG)_7_ RNAs containing 0, 1 or 3 m^1^A.

Supplementary Video 8 Fluorescence recovery after photobleaching (FRAP) of TDP-43–eGFP in the presence or absence of synthetic (CAG)_7_ RNAs containing 0, 1 or 3 m^1^A.

Source Data Source data for Supplementary Figs. 4–8 and 11.

### Source data

Source Data Fig. 1

Source Data Fig. 2

Source Data Fig. 3

Source Data Fig. 4

Source Data Extended Data Fig. 1

Source Data Extended Data Fig. 2

Source Data Extended Data Fig. 3

Source Data Extended Data Fig. 4

Source Data Extended Data Fig. 5

Source Data Extended Data Fig. 6

Source Data Extended Data Fig. 7

## Data Availability

Necessary data for evaluating this study are available in the main text and Supplementary Information. Uncropped gel images are provided in Supplementary Fig. 1. Source data are provided with this paper.
