# Publisher Correction: m^1^A in CAG repeat RNA binds to TDP-43 and induces neurodegeneration

**DOI:** 10.1038/s41586-023-06994-6

**Published:** 2024-01-02

**Authors:** Yuxiang Sun, Hui Dai, Xiaoxia Dai, Jiekai Yin, Yuxiang Cui, Xiaochuan Liu, Gwendolyn Gonzalez, Jun Yuan, Feng Tang, Nan Wang, Alexandra E. Perlegos, Nancy M. Bonini, X. William Yang, Weifeng Gu, Yinsheng Wang

**Affiliations:** 1 Department of Chemistry, University of California Riverside, Riverside, CA USA; 2 Department of Molecular, Cell and Systems Biology, University of California Riverside, Riverside, CA USA; 3 Environmental Toxicology Graduate Program, University of California Riverside, Riverside, CA USA; 4 Center for Neurobehavioral Genetics, The Jane and Terry Semel Institute for Neuroscience and Human Behavior, University of California Los Angeles, Los Angeles, CA USA; 5 Neurosciences Graduate Group, University of Pennsylvania, Philadelphia, PA USA; 6 Department of Biology, University of Pennsylvania, Philadelphia, PA USA; 7 Department of Psychiatry and Biobehavioral Sciences, David Geffen School of Medicine, University of California Los Angeles, Los Angeles, CA USA

**Keywords:** RNA, Molecular neuroscience, Huntington's disease, Neurodegeneration, Biophysical chemistry

Correction to: *Nature* 10.1038/s41586-023-06701-5 Published online 8 November 2023

In the version of the article initially published, there were relabelling errors in Figs. 2b and c and 3c and d, where “(CAG)_22_ + ALKBH3” appeared as “(CAG)_22_ + ALKBH1”. The errors have been corrected in the HTML and PDF versions of the article.

