# Supplementary material for: m1A in CAG repeat RNA binds to TDP-43 and induces neurodegeneration
Source: Nature. 2023 Nov 8;623(7987):580–7. doi: 10.1038/s41586-023-06701-5 (PMC10651481; doi:10.1038/s41586-023-06701-5)
Supplement: Supplementary file 1 — Supplementary Tables 1–4 and Supplementary Figs 1–12. [file 41586_2023_6701_MOESM1_ESM.pdf]

nature portfolio

https://doi.org/10.1038/s41586-023-06701-5

Supplementary information

m¹A in CAG repeat RNA binds to TDP-43 and induces neurodegeneration

In the format provided by the
authors and unedited

Supplementary Tables

Table S1. A list of primer DNA sequences used in this study.

Name	Sequences
ALKBH3 5′ primer-1	5′-ATGGAGGAAAAAAGACGGCGAGCC-3′
ALKBH3 3′ primer-1	5′-TGTATGGTTAGTTAGTACCGAACTGTTTAAACTTACCACCTGTGGGTGACAGGCTG-3′
ALKBH3 5′ primer-2	5′-TCGGTACTAACTAACCATACATATTTAAATTTTCAGTATCTAGGGTCTGTTTGTATCC-3′
ALKBH3 3′ primer-2	5′-GATTAGTTAGTTAGTAAAATCATGTTTAAACTTACCTGTGCCAGTCCACGCTGTC-3′
ALKBH3 5′ primer-3	5′-ATTTTACTAACTAACTAATCTGATTTAAATTTTCAGTGATGATGAACCCTCACTAG-3′
ALKBH3 3′ primer-3	5′-TTCCTTAATCAATTCCGAAACCATCCAGGGTGCCCCTCGAGGGTCTGG-3′
Snb-1 5′ primer	5′-CGGAAATTGTCGGCCGTCGTGGAT-3′
Snb-1 3′ primer	5′-CGGGCTCGCCGTCTTTTTTCCTCCATGTCGTCAAGATGGTCTTATCCGGC-3′
BFP 5′ primer	5′-ATGGTTTCGGAATTGATTAAGGAAA-3′
BFP 3′ primer	5′-GGAATGCTTGAAAGGATCTTGCATCTAGTTGAGCTTGTGTCCGAGCTT-3′
TBB2-3′UTR 5′ primer	5′-ATGCAAGATCCTTTCAAGCATTCC-3′
TBB2-3′UTR 5′ primer	5′-GACTTTTTTCTTGGCGGCACAATA-3′
CAG RNA 5′primer	5′-CCGCAACTCTAGATCATAATCAGC-3′
CAG RNA 3′primer	5′-GCTGCAATAAACAAGTTAACAACAAC-3′

Table S2. A list of synthetic RNA sequences used in this study.

RNA Names	Sequences
Control RNA probe (for quantitative proteomics and EMSA experiments)	5′-biotin-CCGUUCCGCCCAGGCCGCGCCCAGCUGGAAUGCA-3′
m^1A-containing RNA probe (for quantitative proteomics and EMSA experiments)	5′-biotin-CCGUUCCGCCCm^1AGGCCGCGCCCAGCUGGAAUGCA-3′
m^6A-containing RNA probe (for quantitative proteomics and EMSA experiments)	5′-biotin-CCGUUCCGCCCm^6AGGCCGCGCCCAGCUGGAAUGCA-3′
$(CAG)_7$-0m^1A	5′-CAGCAGCAGCAGCAGCAGCAG-3′
$(CAG)_7$-1m^1A	5′-CAGCAGCAGCm^1AGCAGCAGCAG-3′
$(CAG)_7$-3m^1A	5′-Cm^1AGCAGCAGCm^1AGCAGCAGCm^1AG-3′
$(CAG)_{16}$-0m^1A	5′-<br>CAGCAGCAGCAGCAGCAGCAGCAGCAGCAGCAGCAGCAGCAGC<br>AGCAG-3′
$(CAG)_{16}$-3m^1A	5′-<br>CAGCm^1AGCAGCAGCAGCAGCAGCm^1AGCAGCAGCAGCAGCAGC<br>AGCAGCm^1AG-3′

Table S3. A list of shRNAs employed for knocking down genes encoding m^1A methyltransferases in human cells.

**Gene**	**Sequence**
TRMT61A shRNA-1	5′-GAGGCCAGAGGCACCTTATAT-3′
TRMT61A shRNA-3	5′-GCAGATCCTCTACTCCACAGA-3′
TRMT61A shRNA-2	5′-CTCTTAAAGGCTAAGCTTA-3′
TRMT61A shRNA-4	5′-GGCGCAGCTCCATGGTTTATA-3′
TRMT61B	5′-GCGAGGTCATTGTCAGAGATT-3′
TRMT10C	5′-GAGTTAGTTAAACGGTATCAA-3′

Table S4. A list of *C. elegans* strains used in the present study.

**Genotype**	**Strain name**	**Reference**
rmls381[F25B3.3p::Q0::CFP, Topo Pcr2.1] line 1	AM1124	Renee Brielmann lab
rmls172 [F25B3.3p::Q19::CFP]	AM305	Renee Brielmann lab
rmls110 [F25B3.3p::Q40::YFP]	AM101	Caenorhabditis Genetics Center (CGC)
rmls190 [F25B3.3p::Q67::CFP]	AM44	Caenorhabditis Genetics Center (CGC)
rmls190 [F25B3.3p::Q67::CFP]; hdEx1 [snb-1::ALKBH3::BFP::TBB2 3'UTR+rol-6(su1006)]	WG291	In this study
rmIs190 [F25B3.3p::Q67::CFP]; hdEx2 [snb-1::ALKBH3-H257A::BFP::TBB2 3'UTR+rol-6(su1006)]	WG300	In this study

Supplementary Figures

Extended Data Fig. 1

Fig. 1a

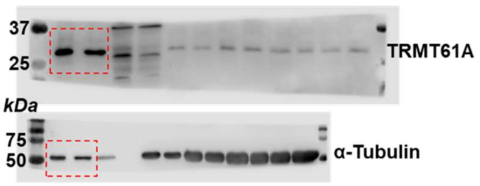

Fig. 1b left panel

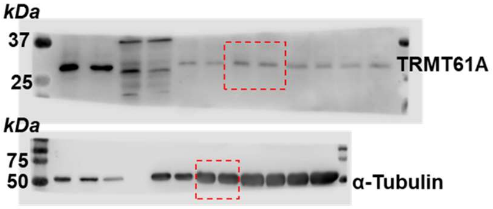

Fig. 1b right panel

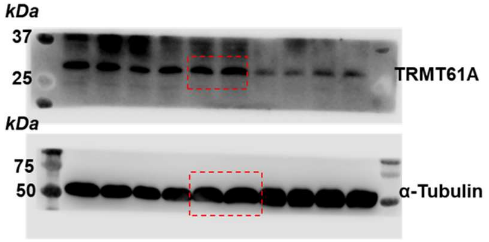

Fig. 1d

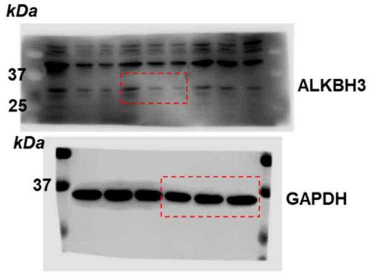

Fig. 1g

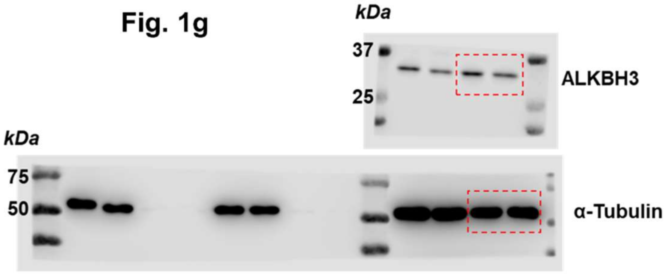

Fig. 1i

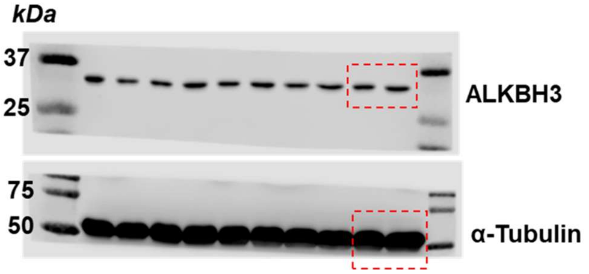

Extended Data Fig. 3

Fig. 3b

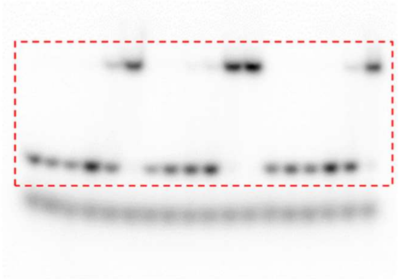

Fig. 3d

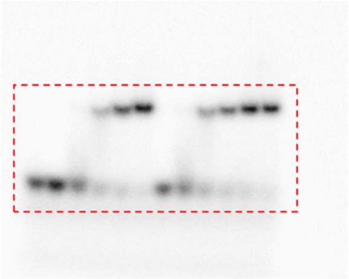

Fig. 3f

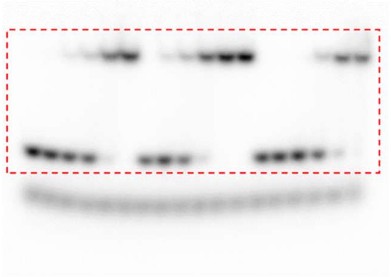

Fig. 3h

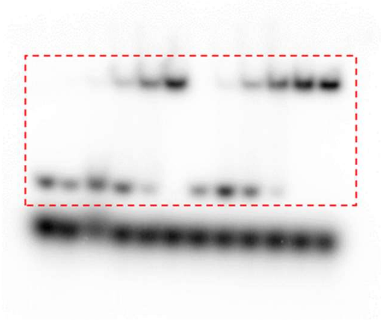

Fig. 3j

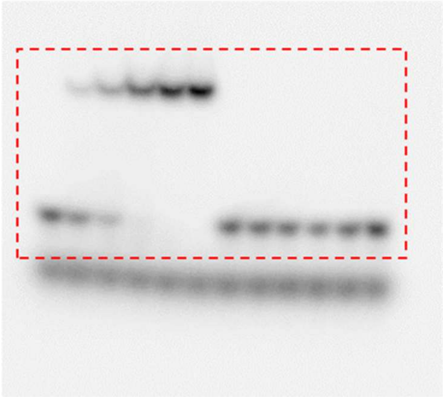

Extended Data Fig. 4

Fig. 4a

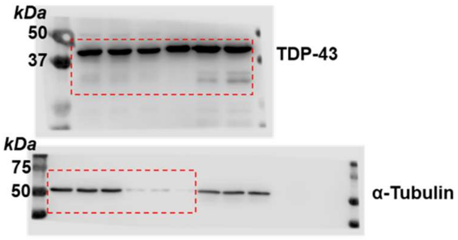

Fig. 4c

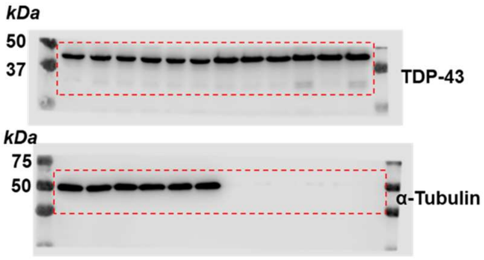

Fig. 4e

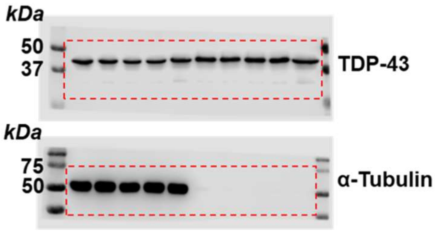

Fig. 4g left panel

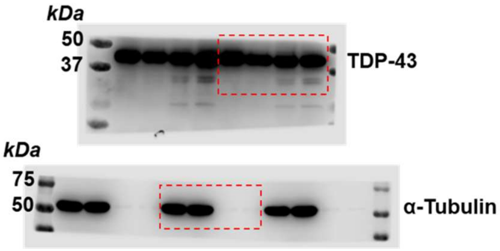

Fig. 4g right panel

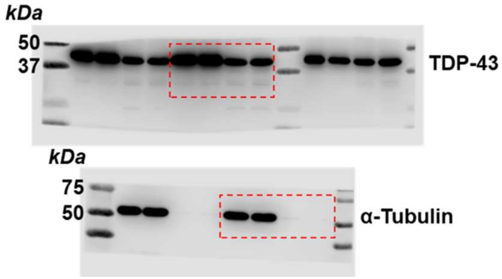

Supplementary Data Fig. S6

Fig. S6a

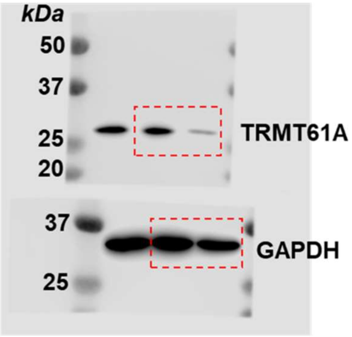

Fig. S6b

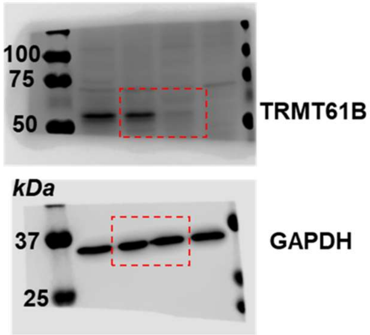

Fig. S6c

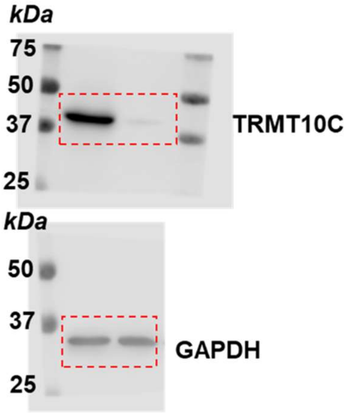

Fig. S6d

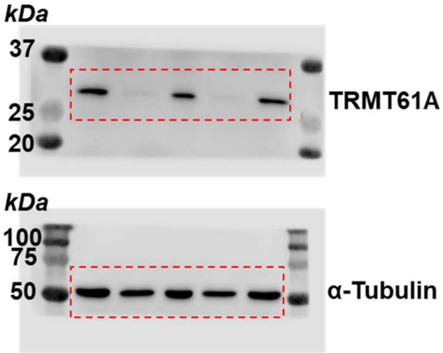

Supplementary Data Fig. S7

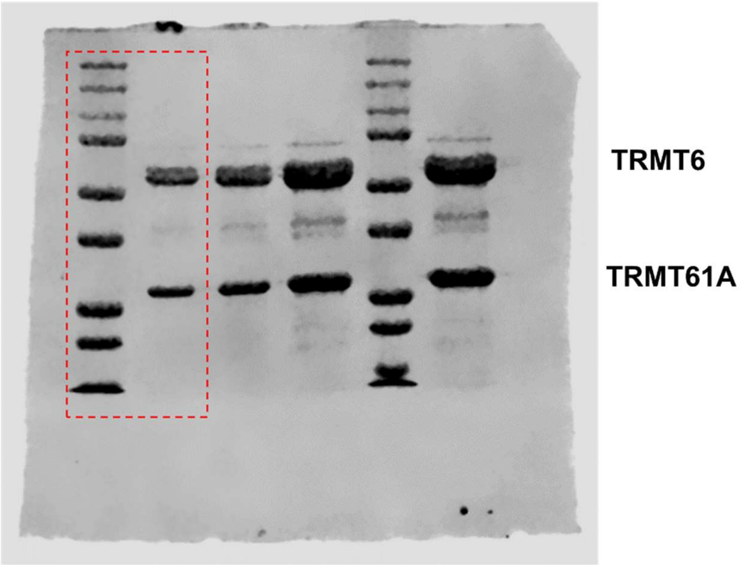

Supplementary Data Fig. S10

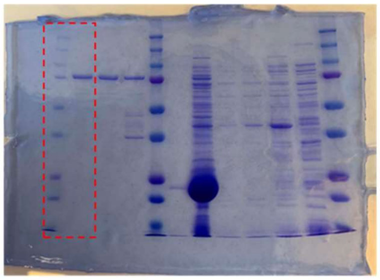

TDP-43-MBP-6xHis

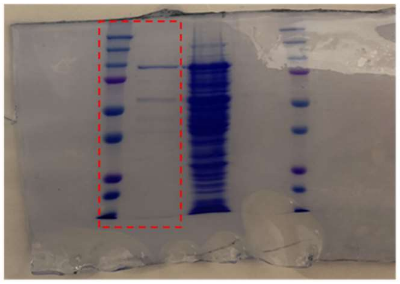

TDP-43 (5FL)-MBP-6xHis

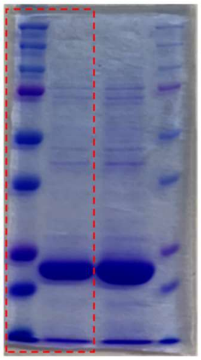

TDP-43 (RRM)-6xHis

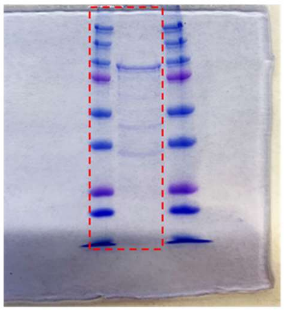

TDP-43-EGFP-MBP-6xHis

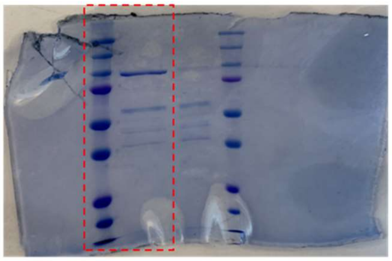

TDP-43 (5FL)-EGFP-MBP-6xHis

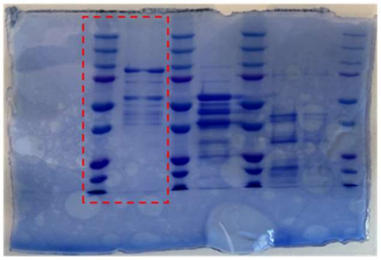

TDP-43 (ΔLCD)-EGFP-MBP-6xHis

Supplementary Data Fig. S11

Fig. S11d

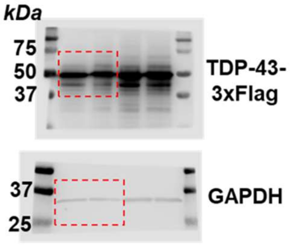

Fig. S11e

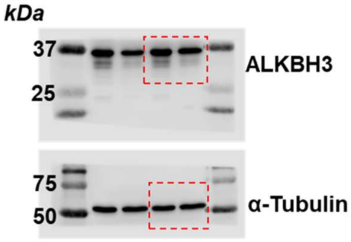

Fig. S11f

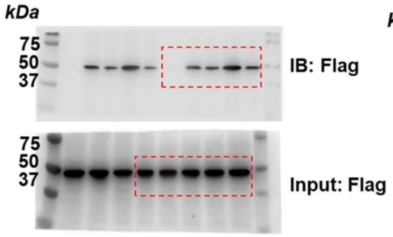

Fig. S11h

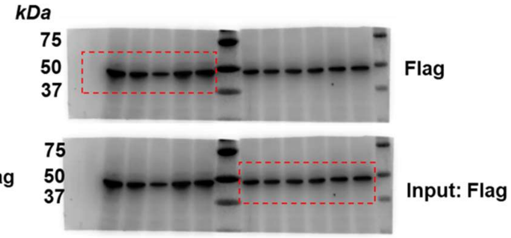

Fig. S11j

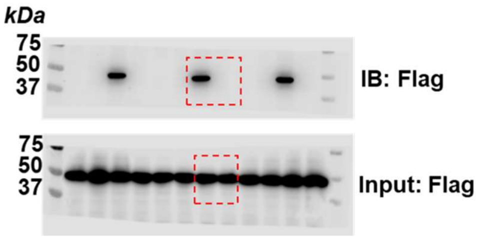

Fig. S1. Uncropped gel images. Cropped regions of the gel images are marked with red boxes.

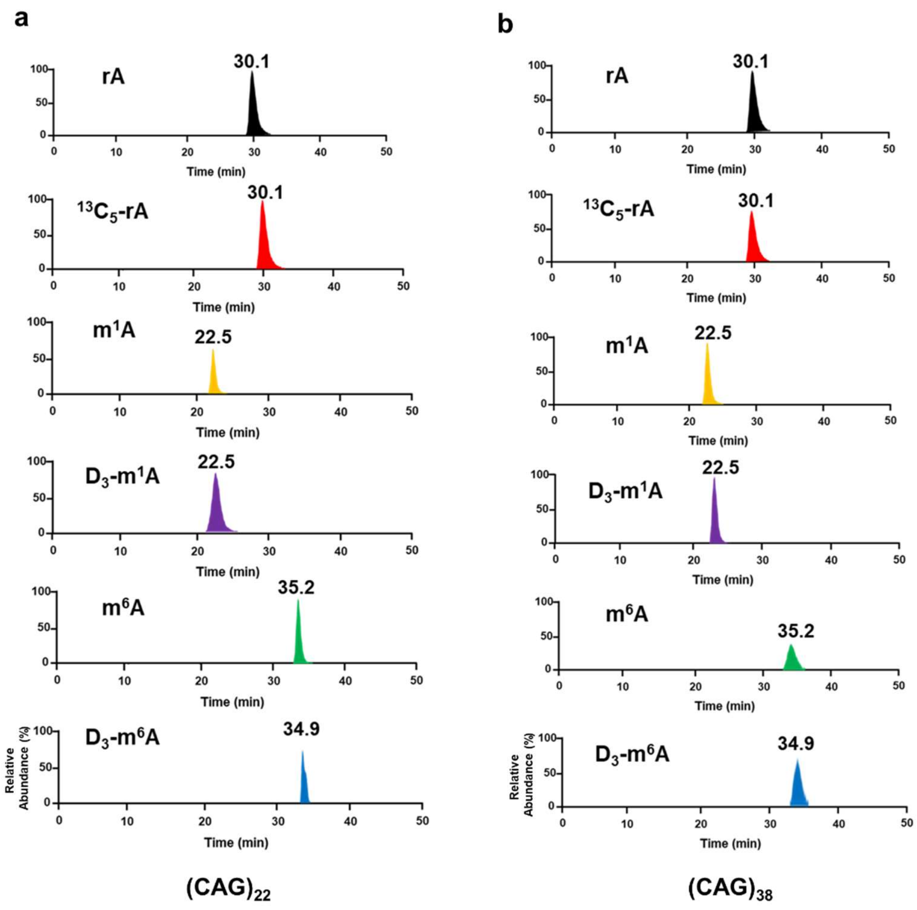

Fig. S2. Representative LC-MS results for the quantifications of rA, m^1A and m^6A in $(CAG)_{22}$ (a) and $(CAG)_{38}$ (b) RNA isolated from HEK293T cells. Shown are the selected-ion chromatograms for monitoring the transition of the neutral loss of a ribose from the protonated ions of the analytes and their corresponding stable isotope-labeled standards.

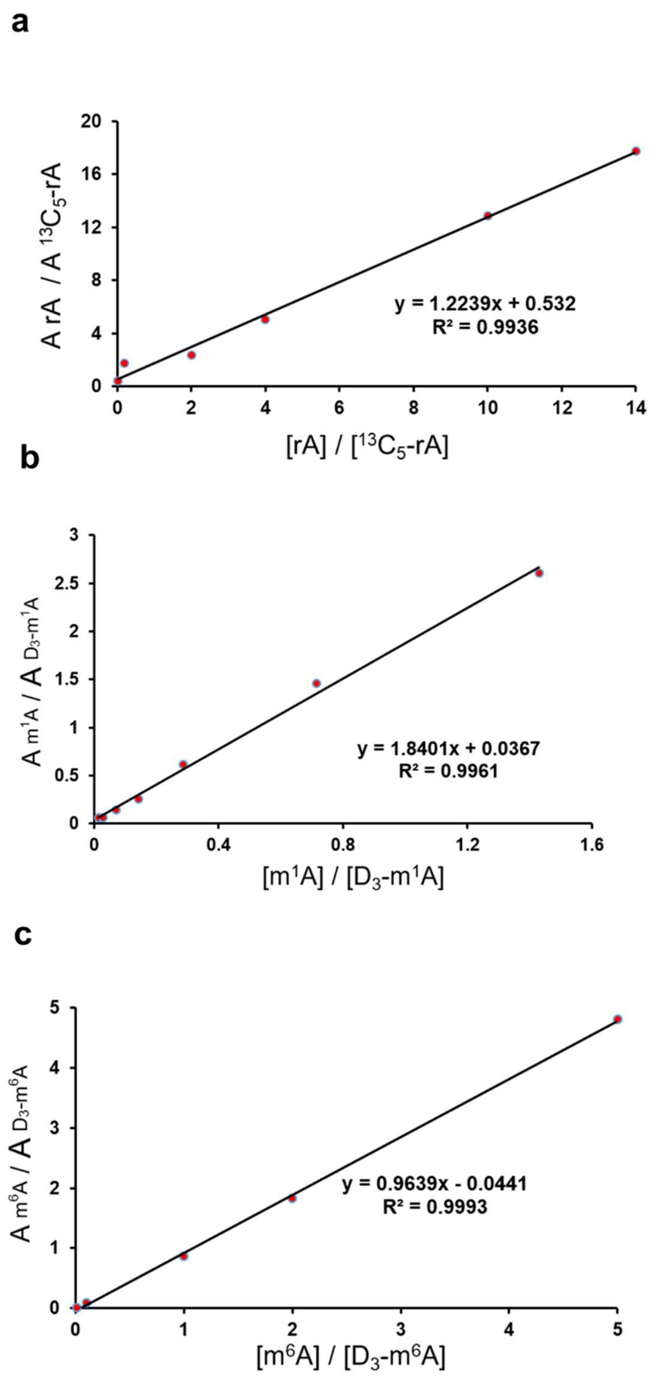

Fig. S3. Calibration curves for the quantifications of rA, m^1A and m^6A. The amounts of internal standards of rA, m^1A and m^6A were 628, 10 and 17 fmol, respectively, and the amounts of unlabeled rA, m^1A and m^6A ranged from 12 fmol-9 pmol, 0.14-14.3 fmol and 0.17-85 fmol, respectively.

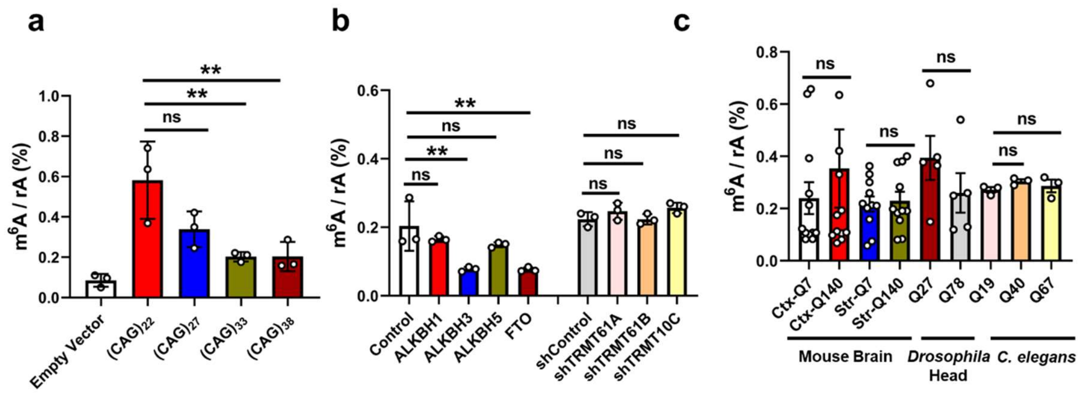

Fig. S4. The ratios of m^{6}A/rA in CAG repeat RNA isolated from cells and tissues. Shown are the m^{6}A/rA ratios CAG repeat RNA isolated from: **a**, HEK293T cells with ectopic expression of different lengths of CAG repeats (n = 3 biologically independent experiments); **b**, HEK293T cells with ectopic expression of $(CAG)_{38}$ RNA and ectopic co-expression of the indicated RNA demethylases or shRNA-mediated stable knockdown of m^{1}A methyltransferases (n = 3 biologically independent experiments); **c**, mouse brain (n = 11 and 12 biologically independent samples for striatum and cortex tissues, respectively), *Drosophila* head (n = 5 biologically independent samples) and *C. elegans* (n = 3 biologically independent experiments). The levels of m^{6}A and rA in CAG repeat RNA were measured by LC-MS/MS, and the frequencies of m^{6}A are expressed as ratios against rA. Data are mean ± s.d. *P* values for the data of striatum or cortex tissues in mouse brain and samples of *Drosophila* head were determined using Two-tailed Student's *t*-test, and one-way ANOVA with Tukey's multiple comparisons test for **a** and **b** and the *C. elegans* data in **c**. ns, $P > 0.05$; $P = 0.0070$ between $(CAG)_{22}$ and $(CAG)_{33}$, and $P = 0.0071$ between $(CAG)_{22}$ and $(CAG)_{38}$ in **a**; $P = 0.0060$ between Control and ALKBH3, and $P = 0.0056$ between Control and FTO in **b**.

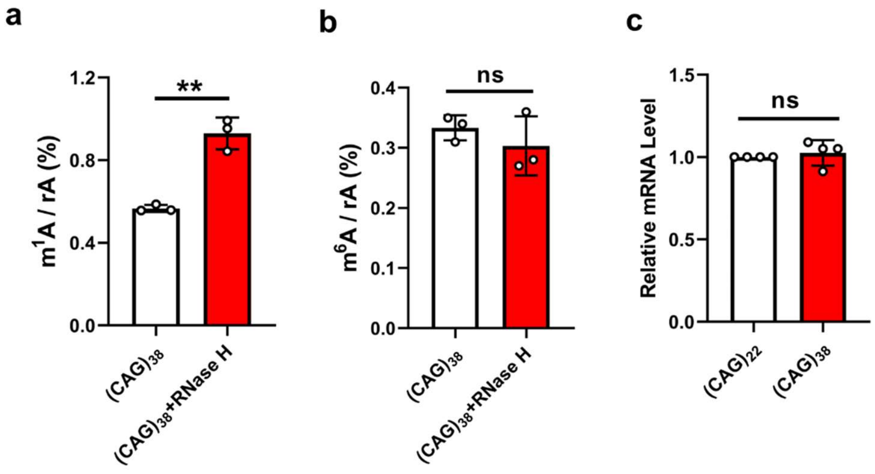

Fig. S5. The ratios of m^1A/rA, but not m^6A/rA, increased in $(CAG)_{38}$ RNA after removal of flanking RNA with RNase H digestion, and RT-qPCR for monitoring the relative levels of expression of $(CAG)_{22}$ and $(CAG)_{38}$ RNA. **a-b**, The levels of m^1A (**a**) and m^6A (**b**) in $(CAG)_{38}$ RNA were measured by LC-MS/MS, and the frequencies of m^1A and m^6A are expressed as their ratios against rA. Data are mean ± s.d., and represent three biologically independent experiments. **c**, RT-qPCR showing similar levels of expression of $(CAG)_{22}$ and $(CAG)_{38}$ mRNA. Data are mean ± s.d. and represent four biologically independent experiments. P values were determined using Two-tailed Student's t-test. ns, $P > 0.05$; $P = 0.0013$ in **a**.

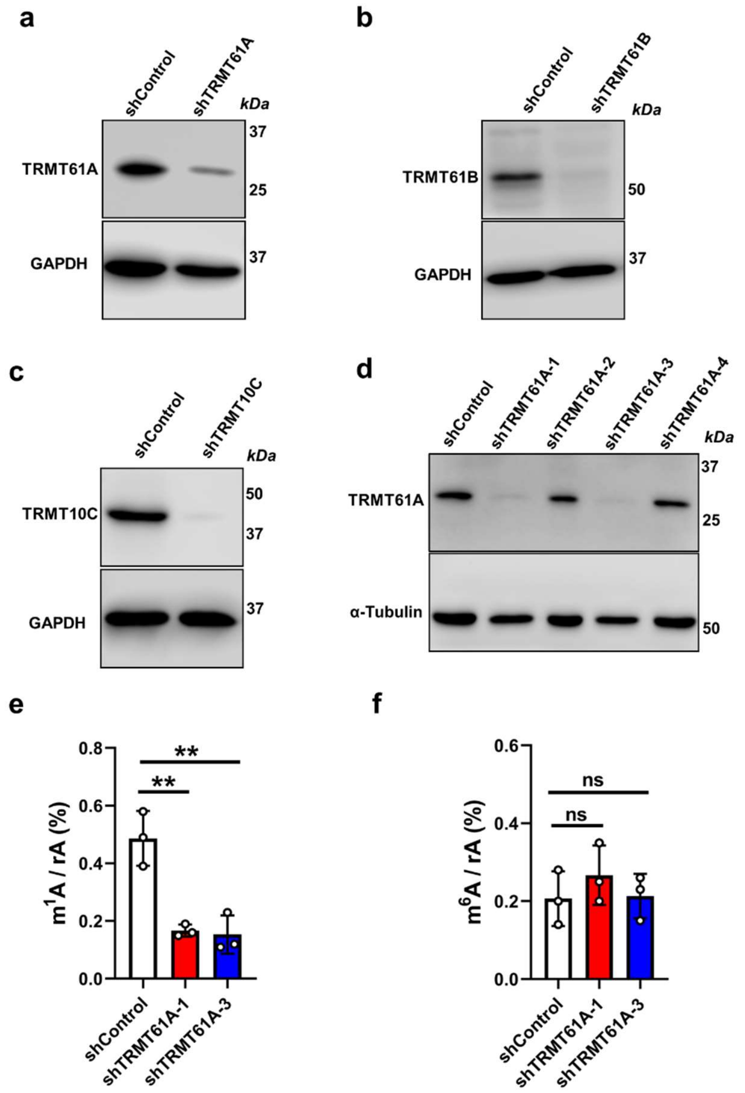

Fig. S6. Knockdown efficiency of genes encoding m^1A methyltransferases in human cells and the ratios of m^1A/rA, but not m^6A/rA, were decreased in U2OS cells upon shRNA-mediated knockdown of TRMT61A. **a-c**, Western blot images showing the shRNA knockdown efficiencies of TRMT61A, TRMT61B, and TRMT10C in HEK293T cells. **d**, Western blot images showing the knockdown efficiencies of TRMT61A in U2OS cells with four different sequences of shRNAs, as determined Western blot analysis; shTRMT61A-1 and shTRMT61A-3 were used for subsequent experiments. The loading control was detected in a separate gel in parallel for **a** and **c**. **e-f**, The levels of m^1A and m^6A in $(CAG)_{38}$ RNA isolated from U2OS cells with or without knockdown of TRMT61A. Data in **e** and **f** are mean ± s.d., and represent three biologically independent experiments. *P* values were determined using one-way ANOVA with Tukey's multiple comparisons test. ns, $P > 0.05$; $P = 0.0029$ between shControl and shTRMT61A-1, and $P = 0.0023$ between shControl and shTRMT61A-3 in **e**. For gel source data, see Supplementary Figure 1.

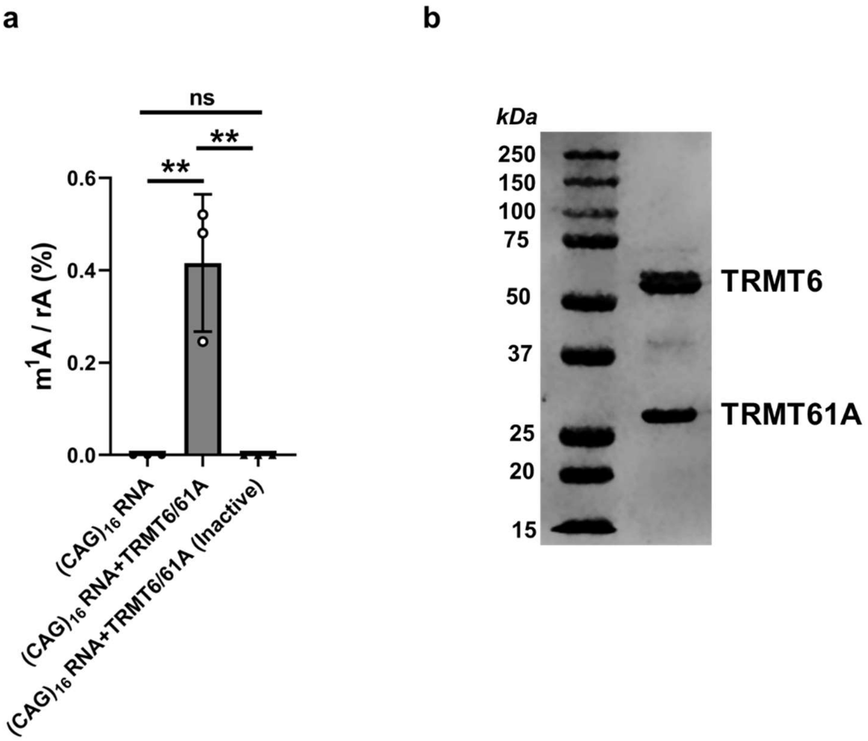

Fig. S7. Recombinant TRMT6/TRMT61A protein complex is capable of installing m^1A in CAG repeat RNA *in vitro*. **a**, The ratios of m^1A/rA in $(CAG)_{16}$ RNA without treatment, or treated with TRMT6/TRMT61A complex, or its heat-inactivated counterpart. Data are mean ± s.d., and represent three biologically independent experiments. *P* values were determined using one-way ANOVA with Tukey's multiple comparisons test. ns, $P > 0.05$; $P = 0.0025$ between $(CAG)_{16}$ RNA and $(CAG)_{16}$ RNA + TRMT6/61A; $P = 0.0025$ between RNA + TRMT6/61A and RNA + TRMT6/61A (Inactive). **b**, SDS-PAGE of the purified TRMT6/TRMT61A recombinant proteins. For gel source data, see Supplementary Figure 1.

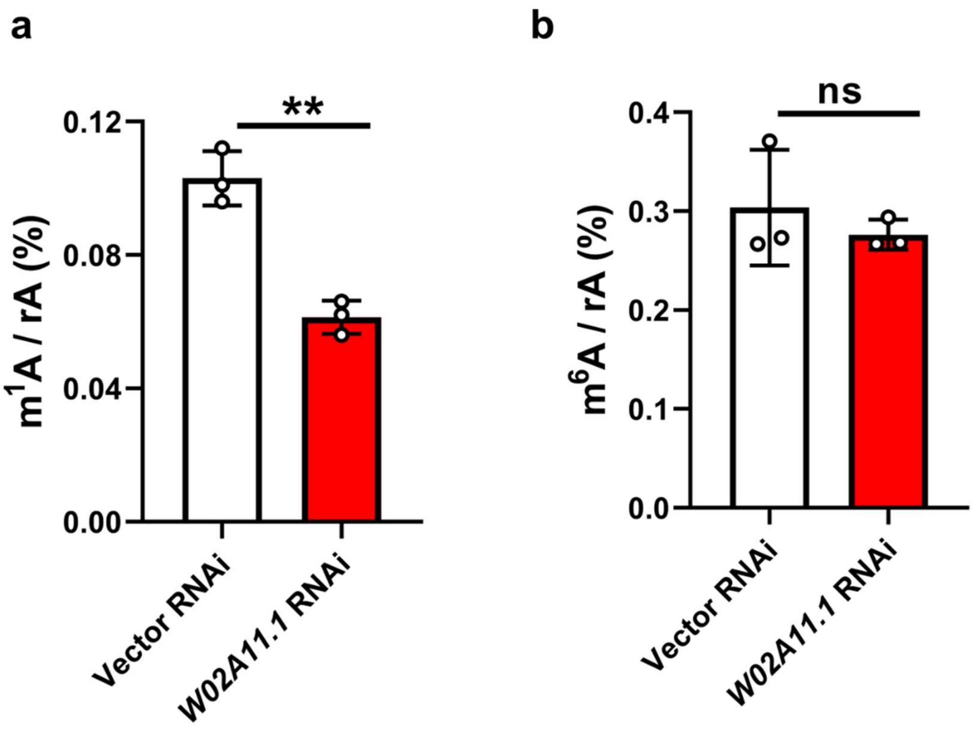

Fig. S8. The ratios of m^1A/rA, but not m^6A/rA, in CAG repeat expansion RNA decrease after knockdown of *W02A11.1* in *C. elegans*. **a-b**, The levels of m^1A and m^6A in Q67 worms with or without knockdown of *W02A11.1*. Data are mean ± s.d., and represent three biologically independent experiments. *P* values were determined using Two-tailed Student's *t*-test. ns, $P >$ 0.05; $^{**}P = 0.0017$ in **a**.

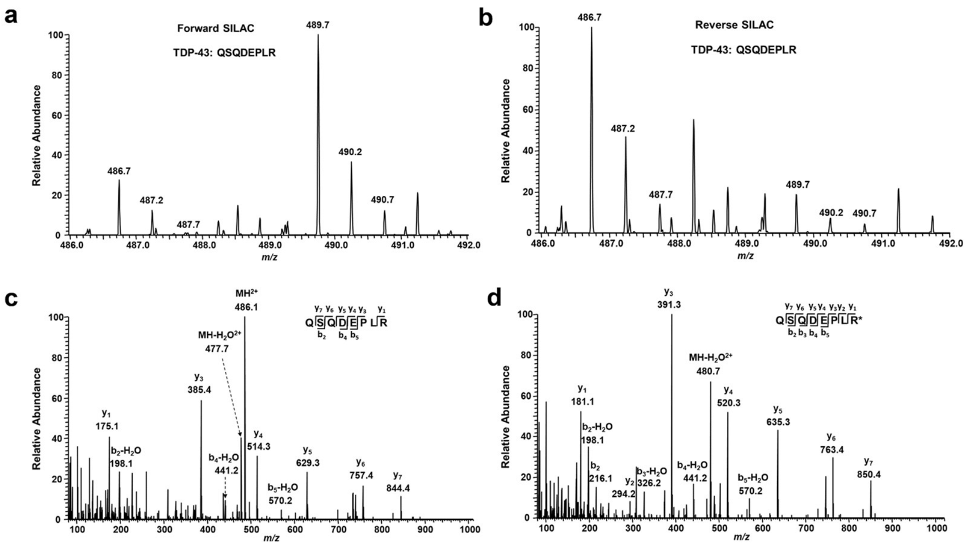

Fig. S9. SILAC-based quantitative proteomic experiment led to the identification of TDP-43 as an m^{1}A-binding protein. We used a 5′-biotin-labeled m^{1}A-carrying RNA sequence derived from human *SOX18* gene, which was previously shown to harbor an m^{1}A site near the start codon in both HEK293T and HeLa cells[23,49], as the probe bait and the corresponding unmethylated sequence as the control bait. We cultured the cells separately in heavy and light culture media, where lysine and arginine were replaced with [$^{13}C_6$,$^{15}N_2$]-L-lysine and [$^{13}C_6$]-L-arginine, respectively, in the heavy culture medium, and extracted proteins from these cells. Equal amount of proteins from the heavy- and light-labeled cells were incubated with biotin-conjugated m^{1}A-bearing RNA and the corresponding unmethylated RNA, respectively, which is designated as the forward SILAC experiments. The opposite incubations were conducted in reverse SILAC experiments to remove experimental bias. After the incubation, the RNA-conjugated beads were extensively washed to remove nonspecific proteins, and the bound proteins were isolated from the beads, digested with trypsin, and analyzed using LC-MS/MS. Shown are the positive-ion ESI-MS (**a-b**) and MS/MS (**c-d**) for a tryptic peptide, QSQDEPLR, derived from TDP-43 from forward (**a**) and reverse (**b**) SILAC experiments.

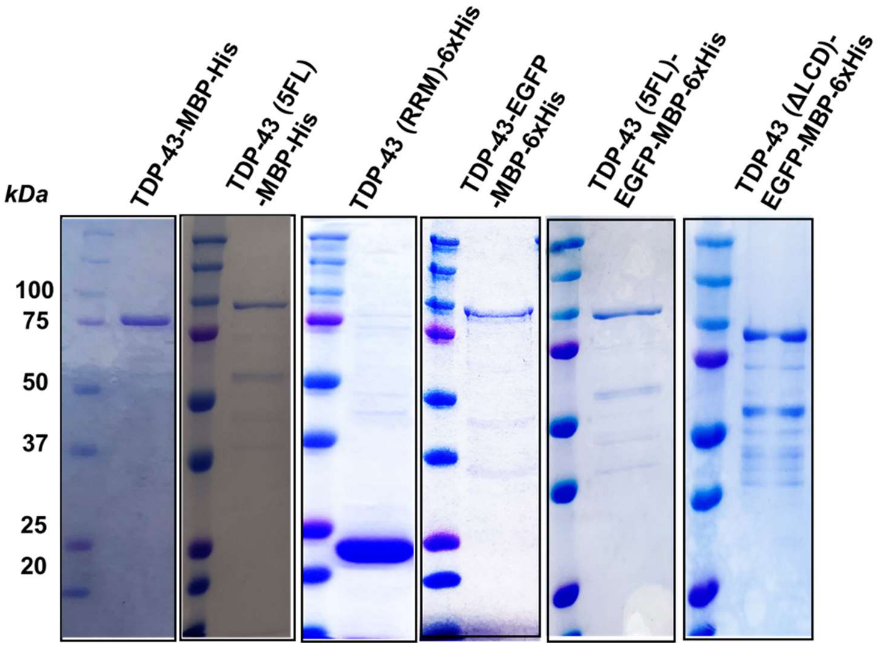

Fig. S10. SDS-PAGE of the indicated TDP-43 proteins tagged with MBP-6×His or EGFP. The experiments for TDP-43-MBP-His and TDP-43-EGFP-MBP-6×His were repeated three times with similar results, other recombinant proteins shown in the image were purified only once. For gel source data, see Supplementary Figure 1.

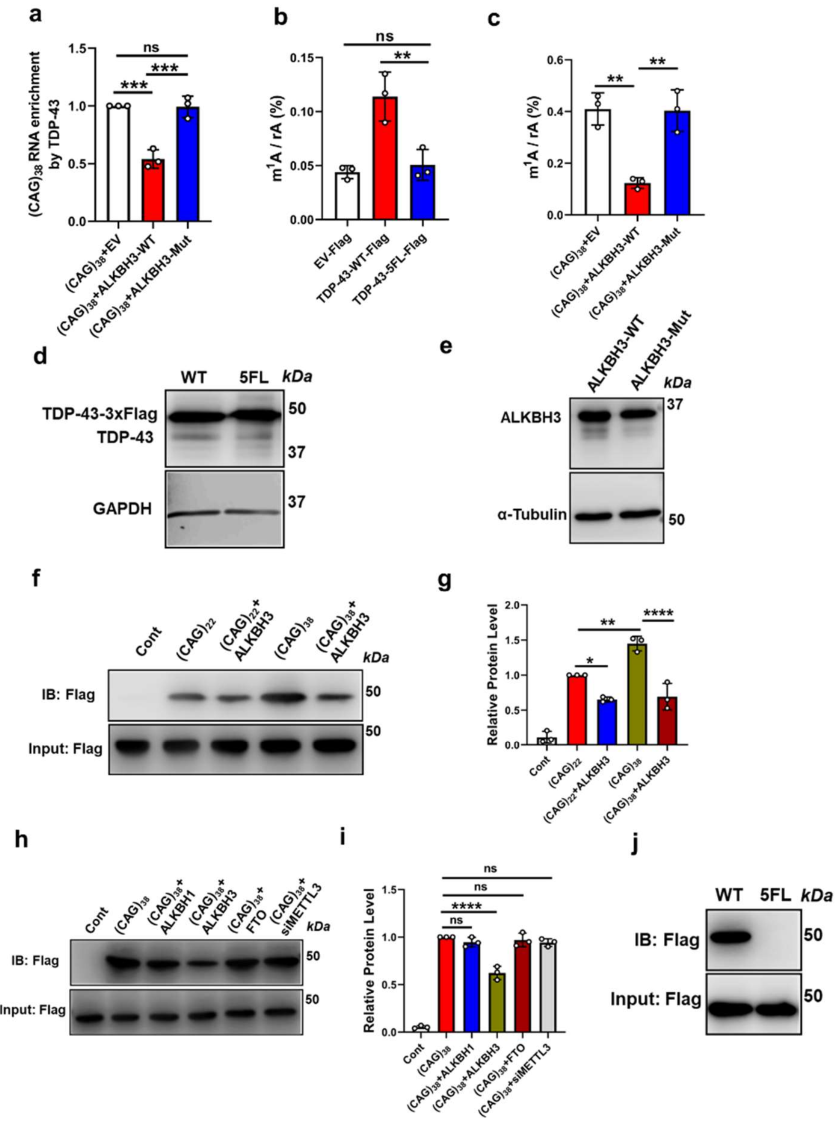

Fig. S11. m[1]A in CAG repeat RNA modulates the interaction between the repeat RNA and TDP-43. **a**, A comparison of the level of $(CAG)_{38}$ mRNA enriched in the pull-down mixtures of TDP-43 with ectopic co-expression of ALKBH3-WT or ALKBH3-Mut, or with empty plasmid control by CLIP-qPCR. **b**, A comparison of the level of m[1]A enriched in the pull-down mRNA mixtures of TDP-43-WT and TDP-43-5FL, or with empty plasmid control. **c**, The levels of m[1]A in $(CAG)_{38}$ repeat RNA isolated from cells with ectopic co-expression of ALKBH3-WT or ALKBH3-Mut, or with empty plasmid control. Data are mean ± s.d., and represent three biologically independent experiments. *P* values were determined using one-way ANOVA with Tukey's multiple comparisons test. ns, $P > 0.05$; $P = 0.00050$ between $(CAG)_{38}$ and $(CAG)_{38}$ + ALKBH3-WT, and $P = 0.00060$ between $(CAG)_{38}$ + ALKBH3-WT and $(CAG)_{38}$ + ALKBH3-Mut in **a**; $P = 0.0065$ between TDP-43-WT-Flag and TDP-43-5FL-Flag in **b**; $P = 0.0026$ for between $(CAG)_{38}$ and $(CAG)_{38}$ + ALKBH3-WT, and $P = 0.0030$ between $(CAG)_{38}$ + ALKBH3-WT and $(CAG)_{38}$ + ALKBH3-Mut in **c**. **d**, Western blot images for monitoring the expression

of TDP-43-WT-3×Flag and TDP-43-5FL-3×Flag. **e**, Western blot images for monitoring the expression of ALKBH3-WT and ALKBH3-Mut. **f-g**, After transient overexpression of TDP-43 in cells expressing $(CAG)_{22}$ and $(CAG)_{38}$ with or without overexpression of ALKBH3 for 48 h, the binding of TDP-43-Flag to CAG repeat RNAs was quantified by Western blot with anti-Flag antibody. Data are mean ± s.d., and represent three biologically independent experiments. *P* values were determined using one-way ANOVA with Tukey's multiple comparisons test. $P = 0.016$ between $(CAG)_{22}$ and $(CAG)_{22}$ + ALKBH3; $P = 0.0026$ $(CAG)_{22}$ and $(CAG)_{38}$; $P < 0.0001$ between $(CAG)_{38}$ and $(CAG)_{38}$ + ALKBH3-WT. **h-i**, The binding of TDP-43 protein to $(CAG)_{38}$ RNA isolated from cells with ectopic co-expression of different demethylase proteins. Data represent three biologically independent experiments. Data are mean ± s.d. *P* values were determined using one-way ANOVA with Tukey's multiple comparisons test. ns, $P > 0.05$; ****$P < 0.0001$. **j**, The levels of TDP-43 WT and TDP-43-5FL bound with $(CAG)_{38}$ RNA. The loading control was detected in a separate gel in parallel for **d**, **f**, **h** and **j**. The experiments shown in **d**, **e** and **j** were repeated three times with similar results. For gel source data, see Supplementary Figure 1.

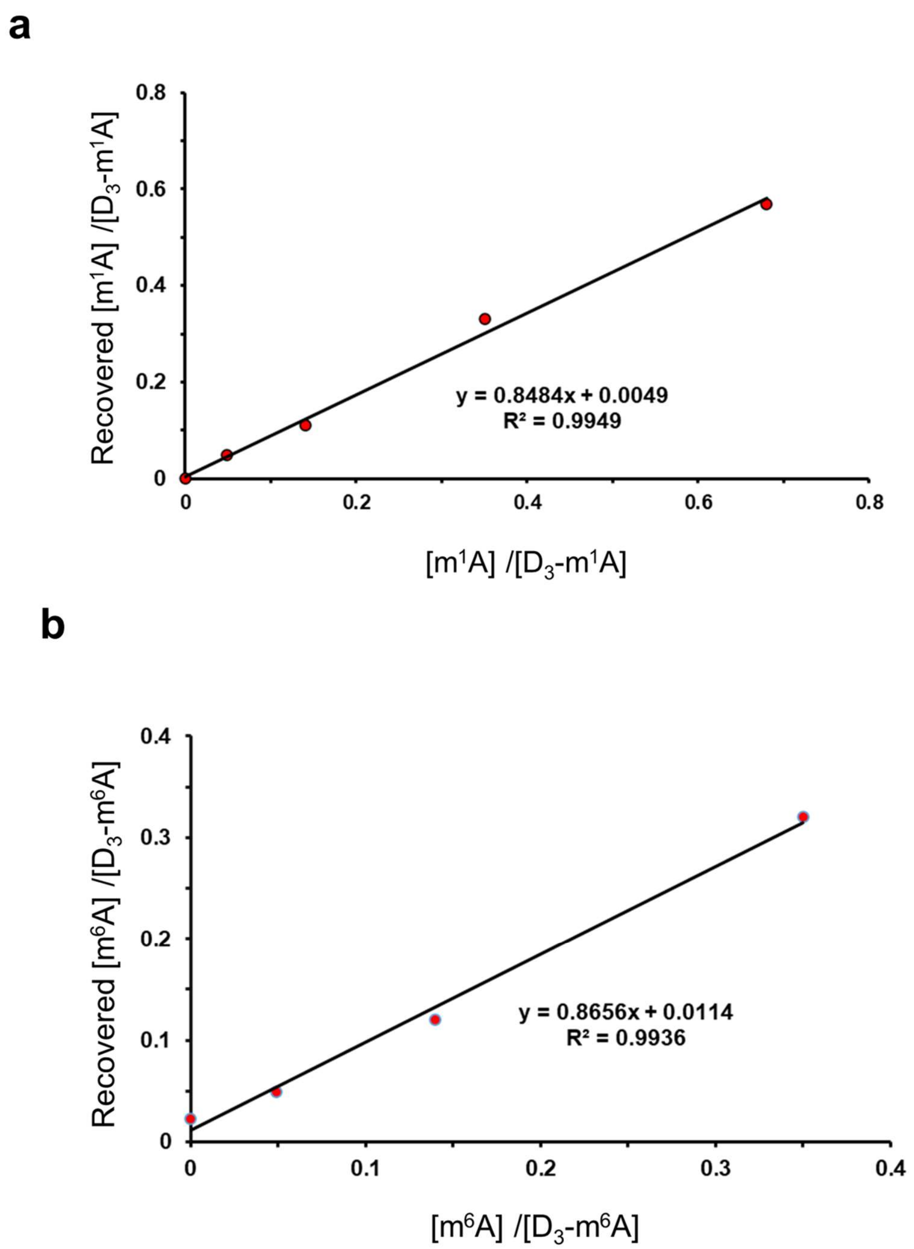

Fig. S12. Recovery calibration curves for the quantifications of m^1A (**a**) and m^6A (**b**) recovered from $(CAG)_7$ RNA spiked with different amounts of $(CAG)_7$-1m^1A and $(CAG)_7$-1m^6A, as determined by enzymatic digestion and LC-MS/MS analyses.
