# Supplementary material for: m1A in CAG repeat RNA binds to TDP-43 and induces neurodegeneration
Source: Nature. 2023 Nov 8;623(7987):580–7. doi: 10.1038/s41586-023-06701-5 (PMC10651481; doi:10.1038/s41586-023-06701-5)
Supplement: Supplementary file 2 — Reporting Summary [file 41586_2023_6701_MOESM2_ESM.pdf]

nature portfolio

Corresponding author(s): Yinsheng Wang

Last updated by author(s): Sep 16, 2023

Reporting Summary

Nature Portfolio wishes to improve the reproducibility of the work that we publish. This form provides structure for consistency and transparency in reporting. For further information on Nature Portfolio policies, see our Editorial Policies and the Editorial Policy Checklist.

Statistics

For all statistical analyses, confirm that the following items are present in the figure legend, table legend, main text, or Methods section.

n/a	Confirmed	
☐	☒	The exact sample size (*n*) for each experimental group/condition, given as a discrete number and unit of measurement
☐	☒	A statement on whether measurements were taken from distinct samples or whether the same sample was measured repeatedly
☐	☒	The statistical test(s) used AND whether they are one- or two-sided<br>*Only common tests should be described solely by name; describe more complex techniques in the Methods section.*
☒	☐	A description of all covariates tested
☒	☐	A description of any assumptions or corrections, such as tests of normality and adjustment for multiple comparisons
☐	☒	A full description of the statistical parameters including central tendency (e.g. means) or other basic estimates (e.g. regression coefficient) AND variation (e.g. standard deviation) or associated estimates of uncertainty (e.g. confidence intervals)
☐	☒	For null hypothesis testing, the test statistic (e.g. *F*, *t*, *r*) with confidence intervals, effect sizes, degrees of freedom and *P* value noted<br>*Give P values as exact values whenever suitable.*
☒	☐	For Bayesian analysis, information on the choice of priors and Markov chain Monte Carlo settings
☒	☐	For hierarchical and complex designs, identification of the appropriate level for tests and full reporting of outcomes
☒	☐	Estimates of effect sizes (e.g. Cohen's *d*, Pearson's *r*), indicating how they were calculated

Our web collection on statistics for biologists contains articles on many of the points above.

Software and code

Policy information about availability of computer code

Data collection	No custom software was used in this study. For Western blot, membranes were scanned using Odyssey Fc Imager (LI-COR). The immunofluoresence data were imaged with an Zeiss 880 Inverted microscope (Zen 2 Blue, Version 2.3).
Data analysis	For statistical analysis, GraphPad Prism version 8.0.1 was used. For image analysis, Image J Fiji was used. For data classification and calculation, Microsoft Excel 2016 was used.

For manuscripts utilizing custom algorithms or software that are central to the research but not yet described in published literature, software must be made available to editors and reviewers. We strongly encourage code deposition in a community repository (e.g. GitHub). See the Nature Portfolio guidelines for submitting code & software for further information.

Data

Policy information about availability of data

All manuscripts must include a data availability statement. This statement should provide the following information, where applicable:

- Accession codes, unique identifiers, or web links for publicly available datasets
- A description of any restrictions on data availability
- For clinical datasets or third party data, please ensure that the statement adheres to our policy

Necessary data for evaluating this study are available in the main text and Supplementary Information. Uncropped gel images are provided in Supplementary Fig. 1. Source data are provided with this paper.

Research involving human participants, their data, or biological material

Policy information about studies with human participants or human data. See also policy information about sex, gender (identity/presentation), and sexual orientation and race, ethnicity and racism.

Reporting on sex and gender	No participants in this study
Reporting on race, ethnicity, or other socially relevant groupings	Not applicable
Population characteristics	Not applicable
Recruitment	Not applicable
Ethics oversight	Not applicable

Note that full information on the approval of the study protocol must also be provided in the manuscript.

Field-specific reporting

Please select the one below that is the best fit for your research. If you are not sure, read the appropriate sections before making your selection.

☒ Life sciences ☐ Behavioural & social sciences ☐ Ecological, evolutionary & environmental sciences

For a reference copy of the document with all sections, see nature.com/documents/nr-reporting-summary-flat.pdf

Life sciences study design

All studies must disclose on these points even when the disclosure is negative.

Sample size	Sample size was chosen to ensure reproducibility of the results. Sample sizes are noted for all experiments. At least 3 independent experiments were carried out for all of the assays and noted in the relevant figure legends.
Data exclusions	No data were excluded.
Replication	All experiments were performed in at least 3 biological replicates, which is indicated in the Figure Legends.
Randomization	Random images of cells were evaluated for immunofluorescence (IF). Randomization for other experiments were not subjective.
Blinding	Blinding is not relevant to the study as the output parameters are not subjective and therefore not subjected to this form of bias.

Reporting for specific materials, systems and methods

We require information from authors about some types of materials, experimental systems and methods used in many studies. Here, indicate whether each material, system or method listed is relevant to your study. If you are not sure if a list item applies to your research, read the appropriate section before selecting a response.

Materials & experimental systems

n/a	Involved in the study	
☐	☒	Antibodies
☐	☒	Eukaryotic cell lines
☒	☐	Palaeontology and archaeology
☐	☒	Animals and other organisms
☒	☐	Clinical data
☒	☐	Dual use research of concern
☒	☐	Plants

Methods

n/a	Involved in the study	
☒	☐	ChIP-seq
☒	☐	Flow cytometry
☒	☐	MRI-based neuroimaging

Antibodies

Antibodies used	TDP-43 (Proteintech, 10782-2-AP, immunoblot, 1:1000; IF, 1:100), G3BP1 (Proteintech, 66486-1-lg, immunoblot, 1:1000; IF, 1:100), TRMT61A (Thermo Fisher, Cat #A305-858A-T, immunoblot, 1:1000), anti-Flag antibody (Cell Signaling Technology, 14793S, immunoblot, 1:2000), ALKBH3 (Cell Signaling Technology, #87620, immunoblot, 1:1000), anti-GAPDH antibody (Santa Cruz

Biotechnology, sc-32233, immunoblot, 1:5000), anti-α-Tubulin antibody (Santa Cruz Biotechnology, sc-32293, immunoblot, 1:5000), Anti-Rabbit IgG (whole molecule)-Peroxidase antibody produced in goat (Sigma, A0545, immunoblot, 1:2500), m-IgGκ BP-HRP (Santa Cruz Biotechnology, sc-516102, immunoblot, 1:2500), Goat anti-Rabbit IgG (H+L) Highly Cross-Adsorbed Secondary Antibody, Alexa Fluor™ 488 (Invitrogen, A-32731, IF, 1:250), Goat anti-Mouse IgG (H+L) Highly Cross-Adsorbed Secondary Antibody, Alexa Fluor™ Plus 647 (Invitrogen, A-32728, IF, 1:250)

Validation

TDP-43 antibody is a rabbit polyclonal antibody recognizing N-terminal domain of TDP-43. Tested Reactivity Human, Mouse, Rat, Zebrafish. Host/Isotype: Rabbit/IgG. Positive WB detected in SH-SY5Y cells, HeLa cells, C2C12 cells, Neuro-2a cells. This antibody has been used in other publications (e.g., Nature, 2021, 594:117-123; Nature, 2022, 603:124-130) according to the Proteintech website.

G3BP1 antibody targets G3BP1 in WB, RIP, IP, IHC, IF, FC, CoIP, ELISA applications and shows reactivity with Human, mouse, rat, pig samples. Host/Isotype: Mouse/IgG.Positive WB detected in LNCaP cells, RAW 264.7 cells, pig brain tissue, HeLa cells, mouse brain tissue, HEK-293 cells, HepG2 cells, Jurkat cells, K-562 cells, HSC-T6 cells, PC-12 cells, NIH/3T3 cells, 4T1 cells. This antibody has been used in other publications (e.g., Nature, 2019, 573:590-594; Nature, 2020, 588:688-692) according to the Proteintech website.

TRMT61A antibody targets TRMT61A in WB and IHC applications and shows reactivity with Human, mouse, rat. Host/Isotype: Rabbit/IgG. Positive WB detected in Jurkat, BT-474, U-251MG, mouse brain, mouse pancreas, mouse spleen, mouse liver, rat testis and rat brain. The specificity of this antibody was verified by shRNA knockdown experiments conducted in this study.

Flag (DYKDDDDK) Tag antibody (D6W5B) Rabbit mAb detects exogenously expressed DYKDDDDK proteins in cells. The antibody recognizes the DYKDDDDK peptide, which is the same epitope recognized by Sigma-Aldrich Anti-FLAG M2 antibody, fused to either the amino-terminus or carboxy-terminus of the target protein. This antibody was applied in WB, IP, IHC, ChIP, eCLIP, IF.This antibody has been used in other publications (e.g., Nature, 2023, 619:819-827; Nat. Commun., 2023, 14:4217) according to the Cell Signaling Technology website.

ALKBH3 antibody targets ALKBH3 in WB, IP, ChIP, IF and IHC applications and shows reactivity with Human, mouse, rat. Host/Isotype: Rabbit/IgG. Positive WB detected in A549 and PC-3 cells. This antibody has been used in other publication (e.g., Cell Death Discov., 2023, 9:159) according to the Cell Signaling Technology website.

GAPDH antibody is recommended for detection of GAPDH of mouse, rat, human, rabbit and Xenopus origin by WB, IP and IF. Host: Mouse. This antibody has been used in other publications (e.g., Nat Commun., 2023, 14:159.; Nucleic Acids Res., 2022, 50:6990-7001) according to the Santa Cruz website.

Anti-alpha Tubulin Antibody (DM1A) is recommended for detection of α Tubulin of mouse, rat, human and avian origin by WB, IP, IF and IHC. Host: Mouse. This antibody has been used in other publications (e.g., Cell, 2023, 186:112-130.; Cell Death Dis., 2023, 14:220)

Goat anti-rabbit IgG-peroxidase conjugate associates with all rabbit Igs. It has been used in western blotting , immunofluorescence staining , immunochemistry and immunoprecipitations. This antibody has been used in other publication (e.g., Journal of Biological Chemistry, 279:47:P49384-49394)

Mouse IgGκ light chain binding protein (m-IgGκ BP) conjugated to horseradish peroxidase (HRP) is a strongly recommended alternative to conventional goat/rabbit anti-mouse IgG secondary antibodies for WB and IHC signal enhancement. This antibody has been used in other publication (e.g., Nat Commun., 2023, 14:1713)

Goat anti-Rabbit IgG (H+L) Highly Cross-Adsorbed Secondary Antibody Alexa Fluor® 488 conjugate was performed to IF analysis. This antibody has been used in other publication (e.g., Development, 2023, 150: dev201376)

Goat anti-Mouse IgG (H+L) Highly Cross-Adsorbed Secondary Antibody, Alexa Fluor™ Plus 647 conjugate was performed to IF analysis. This antibody has been used in other publication (e.g., Nat Commun., 2023, 14:5521)

Eukaryotic cell lines

Policy information about cell lines and Sex and Gender in Research

Cell line source(s)	HEK293T (ATCC, Catalog # CRL-3216) and U2OS (ATCC, Catalog # HTB-96)
Authentication	HEK293T cells and U2OS cells were authenticated by ATCC. Cells were authenticated using using Short Tandem Repeat (STR) analysis as described in 2012 in ANSI Standard (ASN-0002) Authentication of Human Cell Lines.
Mycoplasma contamination	Not contaminated. Tested with LookOut® Mycoplasma PCR Detection Kit (Sigma).
Commonly misidentified lines (See ICLAC register)	None was used in the present study.

Animals and other research organisms

Policy information about studies involving animals; ARRIVE guidelines recommended for reporting animal research, and Sex and Gender in Research

Laboratory animals

Mice were maintained and bred under standard conditions consistent with National Institutes of Health guidelines. The cages were

Laboratory animals	maintained on a 12:12 light/dark cycle, with food and water ad libitum. Brain tissues from wild-type and heterozygous HD knockin Q140 mice in C57BL/6 background at 6-month age were harvested and immediately frozen on dry ice.
Wild animals	No wild animals were used in this study.
Reporting on sex	Brain tissues from both male and female mice were used in this study.
Field-collected samples	No field collected samples were used in the study.
Ethics oversight	The mouse experiments were approved by the University of California, Los Angeles Institutional Animal Care and Use Committee.

Note that full information on the approval of the study protocol must also be provided in the manuscript.
